# Determinants of Divergent Adaptive Immune Responses after Airway Sensitization with Ligands of Toll-Like Receptor 5 or Toll-Like Receptor 9

**DOI:** 10.1371/journal.pone.0167693

**Published:** 2016-12-15

**Authors:** Linda M. Lee, Ming Ji, Meenal Sinha, Matthew B. Dong, Xin Ren, Yanli Wang, Clifford A. Lowell, Sankar Ghosh, Richard M. Locksley, Anthony L. DeFranco

**Affiliations:** 1 Department of Microbiology and Immunology, University of California San Francisco, San Francisco, California, United States of America; 2 Biomedical Sciences Graduate Program, University of California San Francisco, San Francisco, California, United States of America; 3 Department of Laboratory Medicine, University of California San Francisco, San Francisco, California, United States of America; 4 Department of Medicine, University of California San Francisco, San Francisco, California, United States of America; 5 Department of Microbiology and Immunology, College of Physicians and Surgeons, Columbia University, New York, New York, United States of America; 6 Howard Hughes Medical Institute, University of California San Francisco, San Francisco, California, United States of America; Centre National de la Recherche Scientifique, FRANCE

## Abstract

Excessive type 2 helper T cell responses to environmental antigens can cause immunopathology such as asthma and allergy, but how such immune responses are induced remains unclear. We studied this process in the airways by immunizing mice intranasally with the antigen ovalbumin together with either of two Toll-like receptor (TLR) ligands. We found the TLR5 ligand flagellin promoted a type 2 helper T cell response, whereas, a TLR9 ligand CpG oligodeoxyribonucleotide (ODN) promoted a type 1 helper T cell response. CpG ODN induced mRNA encoding interleukin (IL)-12 p40, whereas, flagellin caused IL-33 secretion and induced mRNAs encoding IL-1 and thymic stromal lymphopoietin (TSLP). By using mice deficient in the TLR and IL-1R signaling molecule, myeloid differentiation primary response 88 (MyD88), in conventional dendritic cells (cDCs) and alveolar macrophages (AMs), and by cell sorting different lung populations after 2 hours of *in vivo* stimulation, we characterized the cell types that rapidly produced inflammatory cytokines in response to TLR stimulation. CpG ODN was likely recognized by TLR9 on cDCs and AMs, which made mRNA encoding IL-12. IL-12 was necessary for the subsequent innate and adaptive interferon-γ production. In contrast, flagellin stimulated multiple cells of hematopoietic and non-hematopoietic origin, including AMs, DCs, monocytes, and lung epithelial cells. AMs were largely responsible for IL-1α, whereas lung epithelial cells made TSLP. Multiple hematopoietic cells, including AMs, DCs, and monocytes contributed to other cytokines, including IL-1β and TNFα. MyD88-dependent signals, likely through IL-1R and IL-33R, and MyD88-independent signals, likely from TSLP, were necessary in cDCs for promotion of the early IL-4 response by CD4 T cells in the draining lymph node. Thus, the cell types that responded to TLR ligands were a critical determinant of the innate cytokines produced and the character of the resulting adaptive immune response in the airways.

## Introduction

The mammalian immune system can mount several different types of innate and adaptive responses, each of which are specialized to combat different types of infections. Type 1 immune responses and T helper (T_H_)1 cells promote the host elimination of viruses and intracellular bacteria [1], whereas, type 2 immune responses and T_H_2 cells promote host defense against multi-cellular parasites, such as helminthes and bloodsucking insects, but this response can also cause immunopathology as seen in asthma and allergies [2]. The type of immune response generated in response to pathogens or allergen exposure likely depends on factors including the type of tissue the exposure occurs in, which innate immune receptors are engaged and on which cell types, and which cytokines they produce.

Dendritic cells (DCs) have important roles both in the direct recognition of pathogens and in the initiation of adaptive immune responses [3]. DCs and other cells express pattern recognition receptors (PRRs), which recognize conserved molecules expressed on pathogens. Often, DC recognition of pathogens using one family of PRRs, Toll-like receptors (TLRs), leads to DC production of the cytokine interleukin (IL)-12 [3–5]. IL-12 promotes interferon (IFN)-γ production by various innate lymphocytes [6]. In turn, IFN-γ promotes T_H_1 polarization of activated naïve CD4 T cells. Additionally, DCs take up antigens and migrate from peripheral tissues to secondary lymphoid tissues where they initiate the T cell adaptive immune response [3]. IL-12 made by DCs also acts to stabilize polarization of activated T cells to T_H_1 effectors [6,7]. Alternatively, DCs may respond to cytokines produced by neighboring cells that recognize infection with their PRRs, and these cytokines may induce DC migration to lymphoid tissues and DC initiation of T cells responses [2].

How various types of stimuli lead DCs to induce T_H_2 polarization remains incompletely defined [2]. The cytokines IL-25, IL-33, and thymic stromal lymphopoietin (TSLP) have emerged as important inflammatory cytokines that can drive type 2 immunity. Epithelial cells that are present at environmental interfaces, can produce these cytokines, which then can act on neighboring cell types, including DCs [8,9]. I*n vitro* experiments indicate these cytokines can condition migratory DCs to promote T_H_2 differentiation [10–12]. However, other inflammatory cytokines, such as IL-1α and IL-1β have also been implicated in promoting T_H_2 responses in the lung [13,14]. IL-1α given intranasally (i.n.) activates migratory DCs [13]. IL-1α can be expressed by epithelial and hematopoietic cells [15,16], whereas IL-1β is produced mainly by hematopoietic cells, such as monocytes, macrophages, and DCs [15].

Although innate recognition mechanisms leading to T_H_2 polarization are still incompletely understood, a subset of allergens seem to derive their ability to induce type 2 immunity in the lung from inherent protease activity. Some allergens containing protease activity may act through members of the protease-activated receptor family or through disruption of epithelial barrier function. Other allergens alert the immune system by stimulating PRRs, such as C-type lectin receptors [2,14]. Although TLR stimulation is often associated with T_H_1 responses, it can also promote type 2 immune responses in the lung. For example, house dust mites contain ligands for TLRs, including lipopolysaccharide (LPS), a ligand for TLR4 [17], and contain a protein that can functionally substitute for the TLR4-associated polypeptide MD2 [18]. In addition, flagellin, the TLR5 ligand [19], was found to be present at biologically relevant levels in many house dust extracts [20]. Moreover, TLR ligands such as LPS at low doses [21,22] or flagellin [20,23] are sufficient adjuvants for priming a robust T_H_2 response via i.n. immunization in mice. The T_H_2-inducing effect of flagellin involves contributions of both non-hematopoietic cells [20,23], most likely lung epithelial cells (LECs), and hematopoietic cells [20], but how these cells contribute individually to elicit a T_H_2 response has not been fully defined.

Here, we sought to understand what are the key cell types and cytokines that promote T_H_2 and T_H_1 responses in the lung by characterizing the immune responses when the TLR5 ligand, flagellin, and a TLR9 ligand, CpG motif-containing oligodeoxyribonucleotide (CpG ODN), were used as adjuvants in the lung. Consistent with previous studies [20,23], our current study found flagellin promoted a T_H_2 response. Conversely, we found that CpG ODN induced a robust T_H_1 response, in agreement with previous studies [24,25]. The early cytokine responses to CpG ODN and flagellin had several differences that were important for the subsequent distinctive adaptive immune responses. CpG ODN most likely directly stimulated conventional DCs (cDCs) and alveolar macrophages (AMs) to induce mRNA encoding IL-12 p40. IL-12 was found to be important for innate phase IFN-γ production and T_H_1 polarization. In contrast, flagellin stimulated both hematopoietic cells and LECs to induce synthesis of multiple inflammatory cytokines, including IL-1α, IL-1β, IL-6, TSLP, and TNFα, and release of IL-33. In this response, both MyD88-dependent and MyD88–independent signaling contributed to the activation of DCs, and to their ability to promote subsequent IL-4 production by CD4 T cells. Thus, lung exposure to an antigen in combination with either CpG ODN or flagellin induced distinctly polarized adaptive immune responses due to differences in the cell types responding to these two different TLR ligands, and differences in the inflammatory cytokines they made during the innate phase of the response.

## Results

### Flagellin and CpG ODN promote distinct adaptive immune responses in the lung after i.n. immunization

**Fig 1:**
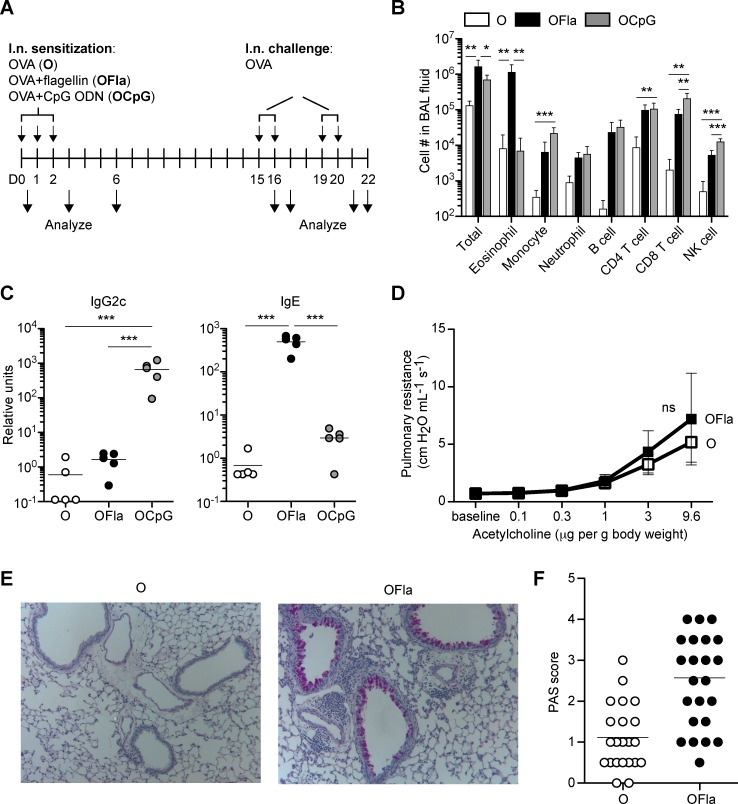
Distinctive adaptive immune responses following i.n. sensitization with OVA plus either flagellin or CpG ODN. **(A)** Outline of i.n. sensitization and rechallenge regimen used. Mice were exposed i.n. to OVA (O), OVA plus flagellin (OFla), or OVA plus CpG ODN (OCpG) on d0, 1, and 2. In some experiments, mice were rechallenged i.n. with OVA two weeks later on d15, 16, 19, and/or 20. Immune responses were assessed at one or more of the time points. For (**B**) and (**C**), mice were administered with OVA, OVA plus 5 μg flagellin, or OVA plus 3 μg CpG ODN i.n., and rechallenged with i.n. OVA. (**B**) Airway inflammation on d22 was assessed by flow cytometry of the cells in the BAL fluid. (**C**) Levels of serum OVA-specific antibodies IgG2c and IgE were measured by ELISA on d21 or d22. For (**D**)-(**F**), the mice were administered with OVA or OVA plus 0.2, 1 or 2 μg flagellin, and rechallenged with OVA. (**D**) The effect of sensitization and rechallenge on pulmonary resistance was measured on d22. (**E**) Representative images of lung sections on d22 stained with Periodic-acid Schiff (PAS) reagent. (**F**) Semi-quantitative PAS scores from lung sections in (**E**). Pulmonary resistance and PAS scores were similar for each dose of flagellin (not shown). Data in (**B**) contain 5 mice per group, and similar results were obtained in two other independent experiments pooled together, data in (**C**) contain 5 mice per group and are representative of at least three independent experiments, data in (**D**) are pooled from four independent experiments with n = 20 for OVA and n = 26 for OVA plus flagellin (except for acetylcholine dose 0.1 μg/g; OVA n = 14, OVA plus flagellin n = 20), and data in (**F**) are pooled from four independent experiments with n = 22 for OVA and n = 27 for OVA plus flagellin. Mice sensitized with OVA plus flagellin had positive PAS scores (average score 2–4) for three of four experiments. Each circle represents one individual mouse. Error bars indicate mean +SD. * P ≤ 0.05, ** P ≤ 0.01, *** P ≤ 0.001 using one-way anova with Bonferroni post-test or Student’s *t*-test in (**D**).

To study how TLR ligands may promote adaptive immune responses in the lung, we slightly modified a well-studied allergic asthma model in which the TLR4 ligand, LPS, was used as an adjuvant in the lung [21,22]. Previous studies showed that immunizing mice with the model antigen ovalbumin (OVA) together with a low dose of LPS resulted in a robust T_H_2 response in the lung, whereas using a higher dose of LPS resulted in a T_H_1 response. We were interested in whether other TLR ligands were able to induce preferentially T_H_1 or T_H_2 responses in a dose-independent manner, unlike the dose-dependent effects of LPS. Previous studies have shown that the TLR5 ligand, flagellin, can induce a T_H_2 response when used as an adjuvant subcutaneously [26,27], and in the lung [20,23]. Conversely, the TLR9 ligand, CpG ODN has been found to promote a T_H_1 response in the lung [24,25], and in other locations [28]. In the regimen we used in this study (Fig 1A), B6 mice were sensitized by i.n. exposure to OVA, or to OVA plus various doses of flagellin or CpG ODN three times on successive days. After 2 weeks, mice were rechallenged with OVA alone for up to four times over a six day time period. Two days after the last i.n. OVA challenge (d22), we found mice sensitized by a TLR ligand as adjuvant had 2-10-fold increased numbers of inflammatory cells in the lung airspace, by assessing cells in the bronchoalveolar lavage (BAL) fluid (Fig 1B). Mice sensitized with OVA plus flagellin had a predominant eosinophil increase in the lung airspace and lung tissue (S1 Fig), using 5 μg and 1 μg flagellin, respectively, consistent with a T_H_2 type of inflammation. In contrast, mice sensitized with OVA plus CpG ODN had few eosinophils in the BAL. Instead, these mice had robust accumulation of CD8 T cells, monocytes, and NK cells, consistent with a T_H_1 type of inflammation. Furthermore, using flagellin as an adjuvant in the lung led to substantial serum titers of OVA-specific IgE, which were not seen when CpG ODN was used as the adjuvant, or when OVA was administered by itself (Fig 1C). In contrast, when CpG ODN was used as an adjuvant, mice produced higher titers of OVA-specific IgG2c. The results seen after OVA challenge are consistent with the notion that flagellin and CpG ODN induced distinctly polarized adaptive immune responses.

Because similar i.n. immunization protocols have been used previously as murine models of asthma, we also examined whether airway hyperresponsiveness (AHR) to acetylcholine and mucus production were present, as these characteristics are increased during T_H_2 inflammation [29]. Mice sensitized with OVA plus flagellin and rechallenged with OVA exhibited a non-significant trend toward greater AHR compared to mice sensitized and rechallenged with OVA alone (Fig 1D). A previous study found a more robust and statistically significant AHR using a somewhat different lung immunization regime with flagellin as adjuvant [20]. The mice sensitized with OVA plus CpG ODN and rechallenged with OVA had similar AHR to mice sensitized and rechallenged with OVA alone (S2 Fig). However, consistent with a previous report [20], we observed that mice sensitized with OVA plus flagellin had substantial production of mucus in the lung, as indicated by Periodic-Acid Schiff staining (Fig 1E and 1F).

**Fig 2:**
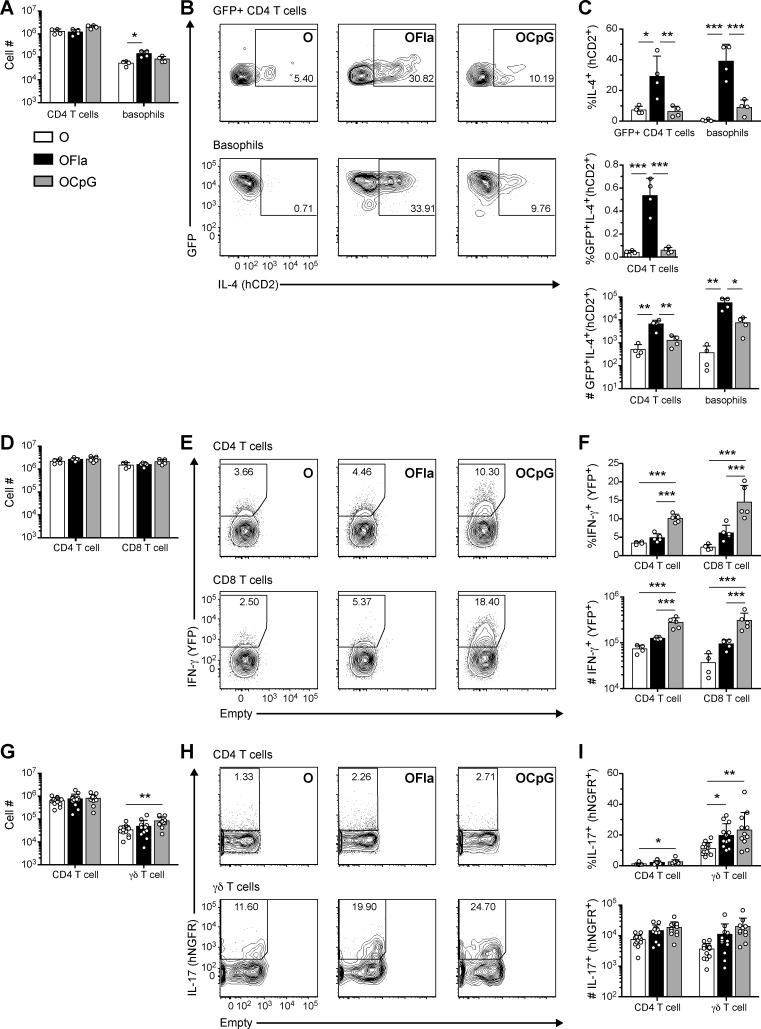
Different polarizations of CD4 T cells in the lung after i.n. sensitization with antigen and either flagellin or CpG ODN. (**A**-**C**) Presence of activated T_H_2 cells and IL-4-producing basophils in the lung of OVA- rechallenged mice. IL-4 reporter (4get/KN2) mice were administered i.n. with OVA, OVA plus 1 μg flagellin, or OVA plus 3 μg CpG ODN, and challenged i.n. with OVA. On d21, expression of IL-4 reporters by lung CD4 T cells and basophils was determined. (**A**) Numbers of lung CD4 T cells and basophils. (**B**) Representative flow cytometry plots of IL-4 production (hCD2^+^) by IL-4 competent (GFP^+^) CD4 T cells (CD1d-tet^-^CD3^+^CD4^+^GFP^+^) and basophils (CD1d-tet^-^CD3^-^CD49b^+^SSC^lo^GFP^+^). (**C**) Percentages IL-4^+^ (hCD2^+^) of IL-4 competent (GFP^+^) CD4 T cells and basophils, percentages GFP^+^IL-4^+^(hCD2^+^) of total CD4 T cells, and numbers of GFP^+^IL-4^+^(hCD2^+^) CD4 T cells and basophils. (**D**-**F**) Presence of activated T_H_1 cells and IFN-γ-expressing CD8 T cells in the lungs of rechallenged mice. IFN-γ reporter (GREAT) mice were administered i.n. with OVA, OVA plus 5 μg flagellin, or OVA plus 3 μg CpG ODN, and challenged with i.n. OVA. On d16, expression of IFN-γ reporter by lung CD4 and CD8 T cells was determined. (**D**) Numbers of lung CD4 and CD8 T cells. (**E**) Representative flow cytometry plots of IFN-γ (YFP^+^) by CD4 and CD8 T cells. (**F**) Percentages and numbers of IFN-γ^+^ CD4 and CD8 T cells. (**G**-**I**) Presence of activated T_H_17 cells and IL-17A-expressing γδ T cells in the lungs of rechallenged mice. IL-17 reporter (SMART-17A) mice were administered i.n. with OVA, OVA plus 1 μg flagellin, or OVA plus 3 μg CpG ODN, and challenged with i.n. OVA. On d17, expression of the IL-17 reporter by lung CD4 and γδ T cells was determined. (**G**) Total numbers of lung CD4 and γδ T cells. (**H**) Representative flow cytometry plots of IL-17 (hNGFR^+^) expression by CD4 and γδ T cells. (**I**) Percentages and numbers of hNGFR^+^ CD4 and γδ T cells. Data in (**A**-**C**) contain four mice per group and are representative of one of three independent experiments, data in (**D**-**F**) contain 4–5 mice per group and are representative one of two independent experiments, data in (**G**-**I**) are pooled from three independent experiments with combined totals of 11–13 mice per group. Each circle represents one individual mouse. Error bars indicate mean + SD. * P ≤ 0.05, ** P ≤ 0.01, *** P ≤ 0.001 using one-way anova with Bonferroni post-test.

To directly address which types of T helper cells were being induced, we examined cytokine production in CD4 T cells present in lung tissue after rechallenge with OVA by using cytokine reporter mice. The advantages of this approach are that the *in vivo* response is being measured, and that the cytokine secretion of other immune cells can also be assessed. In the 4get/KN2 IL-4 reporter mice (*Il4*^*4get/KN2*^), cells that have opened up the IL-4 gene locus express green fluorescent protein (GFP) from the *Il4*^*4get*^ allele [30,31], and cells that have recently produced IL-4 additionally express cell surface human CD2 from the *Il4*^*KN2*^ allele [32]. In GREAT IFN-γ reporter mice (*Ifng*^*GREAT/GREAT*^), cells that have recently produced IFN-γ express yellow fluorescent protein (YFP) [33]. In the SMART-17A IL-17A reporter mice (*Il17*^*SMART/SMART*^), cells that have recently produced IL-17A express cell surface human nerve growth factor receptor (hNGFR) [34]. We first examined at which time point we could optimally detect cytokine reporter positive cells in the lung in the three different reporter lines after i.n. OVA challenge. We found the optimal time points to detect IL-4 reporter, IFN-γ reporter, and IL-17-reporter positive cells were one day after the fourth, first, or second i.n. OVA challenge, respectively (data not shown). Although after sensitization with either OVA alone, OVA plus flagellin, or OVA plus CpG ODN, the numbers of CD4 T cells present in the lung tissue were similar (Fig 2A, 2D and 2G) at all time points examined, the *in vivo* production of cytokines by the effector T cells in those mice was quite different. Flagellin used as adjuvant induced a greater number and percentage of IL-4-producing (GFP^+^hCD2^+^) CD4 T cells in the lung (Fig 2B and 2C) than did CpG ODN as adjuvant. In contrast, CpG ODN used as adjuvant induced greater numbers and percentages of IFN-γ-producing (YFP^+^) CD4 T cells and CD8 T cells in the lung (Fig 2E and 2F) than flagellin used as adjuvant. Interestingly, i.n. sensitization with flagellin also led to increased numbers of basophils in the lung, many of which produced IL-4 (Fig 2A–2C). In contrast, immunizations with either flagellin or CpG ODN as adjuvant followed by rechallenge with OVA led to small increases in the percentages of IL-17-producing (hNGFR^+^) CD4 T cells and γδ T cells in the lung (Fig 2H and 2I). These data demonstrate that i.n. immunization with flagellin as adjuvant and subsequent rechallenge with antigen led to a predominant T_H_2 response in the lung, whereas immunization with CpG ODN as adjuvant and rechallenge led to a predominant T_H_1 response in the lung. These results are in agreement with the different cellular compositions of the inflammatory infiltrates and antibody isotypes. Our findings are consistent with previous studies showing i.n. administration OVA plus flagellin led to elevated IgE and eosinophil levels [20], and production of T_H_2 cytokines by *in vitro* antigen-stimulated LN cells [23] and T cells [20].

### Flagellin and CpG ODN induce robust innate inflammatory infiltrates in the lung

**Fig 3:**
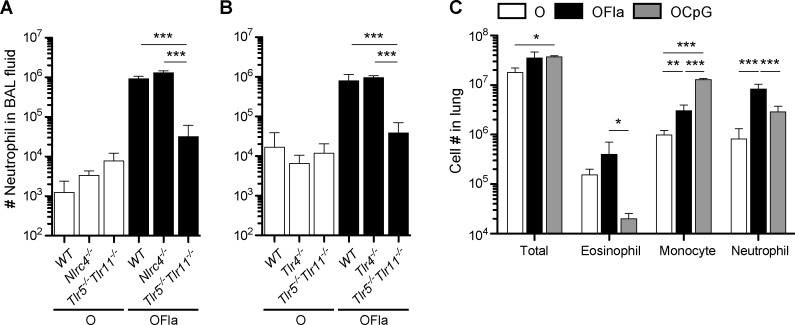
Flagellin and CpG ODN induce robust innate inflammatory infiltrates in the lung. (**A**), (**B**) Neutrophil accumulation in the airways one day after a single i.n. administration (d1) of OVA (O) or OVA plus flagellin (1 μg) (OFla) in wildtype, *Tlr5*^*-/-*^*Tlr11*^*-/-*^, and *Nlrc4*^*-/-*^ mice (**A**) or in wildtype, *Tlr5*^*-/-*^*Tlr11*^*-/-*^ and *Tlr4*^*-/-*^ mice (**B**), as assessed by flow cytometry of the cells in the BAL fluid. (**C**) Cellular composition of the innate inflammatory infiltrate in the lung one day after the third i.n. sensitization (d3) with OVA, OVA plus flagellin (1 μg), or OVA plus CpG ODN (3 μg) (OCpG). Data in (**A**) contain 4 mice per group and are representative of two independent experiments, data in (**B**) contain 4 mice per group and are representative of two independent experiments, and data in (**C**) contain 3–4 mice per group and are representative of three independent experiments. Error bars indicate mean +SD. * P ≤ 0.05, ** P ≤ 0.01, *** P ≤ 0.001 using one-way anova with Bonferroni post-test. In (**A**) and (**B**), all groups of OVA-treated mice have statistically significant different values compared to OVA plus flagellin-treated wild-type mice (**A** and **B**), *Nlrc4*^*-/-*^ (**A**), and *Tlr4*^*-/-*^ mice (**B**) (*** P ≤ 0.001 for all comparisons; not indicated on the panel).

As the i.n. administration of flagellin or CpG ODN resulted in distinct adaptive immune responses upon lung rechallenge with the antigen, we wondered whether the innate inflammatory responses induced by these adjuvants in the lung were also distinct. Flagellin promoted a robust influx of neutrophils in the lung airspace one day after the first i.n. administration (Fig 3A and 3B), which is consistent with previous studies [35–37]. Flagellin can be recognized extracellularly by TLR5 and intracellularly by nucleotide-binding domain, leucine-rich repeat-containing (NLR) proteins Naip5 and Naip6 of the NLRC4 inflammasome [38]. Because previous studies showed that flagellin may be recognized by TLR11 in some circumstances [39], we examined neutrophil influx in *Tlr5*^*-/-*^*11*^*-/-*^ mice. The neutrophil influx after flagellin i.n. administration was almost completely abrogated in *Tlr5*^*-/-*^*11*^*-/-*^ mice (Fig 3A and 3B), but remained intact in *Nlrc4*^*-/-*^ mice (Fig 3A), indicating that the adjuvant effect of flagellin was mainly dependent on TLR5 and/or TLR11 signaling. Previous studies have similarly reported that the adjuvant effect of inhaled flagellin in the lung is dependent on TLR5 [20,23,36] and independent of NLRC4 [20,23]. In addition, the neutrophil influx after flagellin i.n. administration remained intact in *Tlr4*^*-/-*^ mice (Fig 3B), indicating that contamination of this preparation of flagellin with LPS, a ligand for TLR4, was minimal.

Similarly, both flagellin and CpG ODN caused the robust accumulation of inflammatory cells in the lung one day after the third i.n. administration of OVA plus TLR ligand (Fig 3C). CpG ODN led to increased numbers of monocytes, as did flagellin to a lesser extent. In contrast, flagellin induced a larger number of neutrophils in the lung on d3 than did CpG ODN. Although i.n. sensitization of mice with OVA plus flagellin and repeated rechallenge with OVA led to robust accumulation of eosinophils in the lung airspace on d22, the early inflammatory infiltrate induced by OVA plus flagellin did not include a substantial number of eosinophils in the lung greater than what was seen in mice treated with OVA alone.

Because i.n. administration of OVA plus flagellin and, to a lesser extent, OVA plus CpG ODN induced a robust infiltration of neutrophils in the lung, we also examined IL-17A production during the innate phase of the response by using the SMART-17A cytokine reporter mice. Flagellin treatment led to increased percentages of IL-17A-producing γδ T cells and CD4 T cells on d3 (S3 Fig). CpG ODN also promoted an increase, but typically to a lesser degree.

### The CpG ODN-induced type 1 immune response is dependent on IL-12 and on MyD88 signaling in cDCs and/or AMs

**Fig 4:**
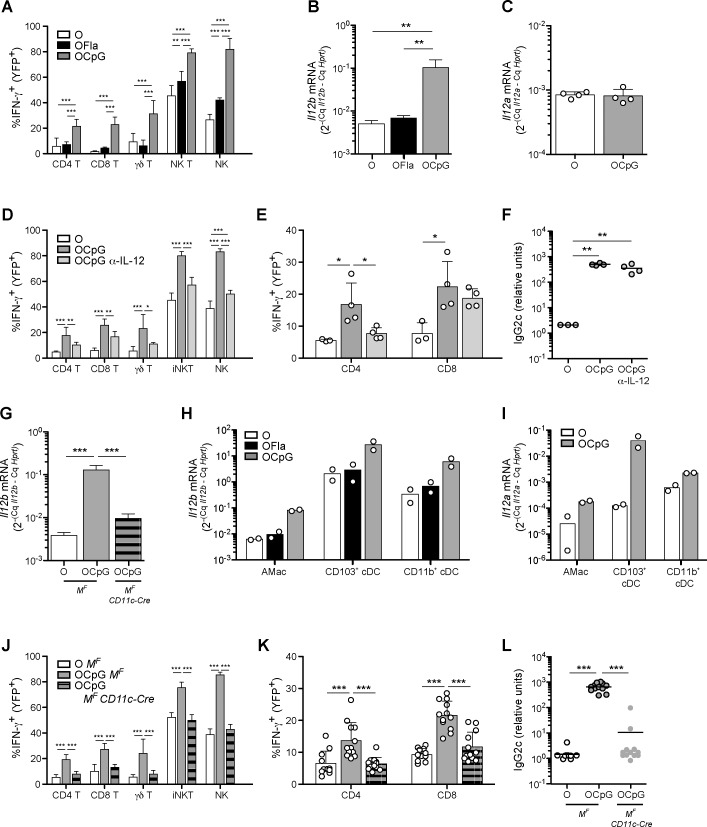
The CpG ODN-induced type 1 immune response is dependent on IL-12 and MyD88 signaling in cDCs and/or AMs. (**A**) Percentages of CD4 T cells, CD8 T cells, γδ T cells, NK T cells, and NK cells producing IFN-γ (YFP^+^) in GREAT reporter mice one day after third i.n. administration (d3) of OVA, OVA plus flagellin (5 xg), or OVA plus CpG ODN (3 μg). (**B**) *Il12b* and (**C**) *Il12a* mRNA induction in whole lung tissue 2 h after one i.n. administration of OVA, OVA plus flagellin (1 μg), or OVA plus CpG ODN (3 μg) in wild-type mice as measured by qPCR. Samples were normalized to *Hprt* mRNA. (**D-F**) GREAT reporter mice were treated with anti-IL-12 p40 or with control antibody (rat IgG2a), one day before initial sensitization (700 μg intraperitoneally (i.p.)) and again on d2 (300 μg i.p.) (**E, F**). These mice were sensitized i.n. with OVA or OVA plus CpG ODN (0.75 μg) on d0, 1 and 2 and rechallenged with OVA alone on d15. (**D, E**) Percentages of IFN-γ (YFP^+^) lymphocytes in the lung on d3 (**D**) and d16 (**E**). (**F**) Serum levels of OVA-specific IgG2c on d16. (**G**) *Il12b* mRNA induction in whole lung tissue 2 h after one i.n. administration of OVA, or OVA plus CpG ODN (3 μg) in *Myd88*^*fl/fl*^ mice (*M*^*F*^) or *Myd88*^*fl/fl*^*CD11c-Cre* (*M*^*F*^ *CD11c-Cre*) as measured by qPCR. Samples were normalized to *Hprt* mRNA. **(H-I)** Inductions of *Il12b* (**H**) and *Il12a* (**I**) mRNAs in sorted cell populations from lung tissue 2 h after i.n. treatment as in (**B**). (**J-L**) *M*^*F*^ or *M*^*F*^ *CD11c-Cre* mice expressing the GREAT reporter were sensitized i.n. with OVA or OVA plus 0.75 μg CpG ODN on d0, 1, and 2, and rechallenged on d15 with OVA alone. (**J, K**) Percentages of IFN-γ reporter^+^ lymphocytes in the lung on d3 (**J**) and on d16 (**K**). (**L**) Serum levels of OVA-specific IgG2c on d16. Data in (**A**) are pooled from two independent experiments with combined totals of 7–8 mice per group, data in (**B**) contain 5 mice per group and are representative of two independent experiments, data in (**C**) contain 4 mice per group and are representative of two independent experiments, data in (**D**) are pooled from two independent experiments with 6–7 mice per group, and similar results were obtained in a third independent experiment, data in (**E, F**) contain 3–4 mice per group and are representative of one of three independent experiments, data in (**G**) contain 4 mice per group and are representative of two independent experiments, data in (**H, I**) are pooled from two independent experiments with combined totals of 6–7 mice per group, data in (**J**) are pooled from two independent experiments with combined totals of 5 or 8 mice per group, and data in (**K, L**) are pooled from three independent experiments with combined totals of 10 or 12 mice per group. Each circle represents one individual mouse except in (**H, I**), in which each circle represents 3–4 mice pooled before cell sorting. Error bars indicate mean +SD. * P ≤ 0.05, ** P ≤ 0.01, *** P ≤ 0.001 using one-way anova with Bonferroni post-test.

Because previous studies have shown that TLR stimulation in DCs can promote IL-12 production, and that IL-12 is a potent inducer of IFN-γ in lymphocytes [6], we also examined IFN-γ production during the innate immune response. Compared to i.n. administration of OVA alone, OVA plus CpG ODN led to increased production of IFN-γ by several different lymphocyte populations (Fig 4A). CpG ODN treatment also induced a robust increase in mRNA encoding the p40 subunit of IL-12 (*Il12b*) in the lung (Fig 4B). We observed no difference in mRNA encoding the p35 subunit of IL-12 (*Il12a*) (Fig 4C). However, *Il12a* induction after i.n. OVA plus CpG ODN treatment in some cell types may have been obscured in this analysis using whole lung tissue, as some cell types constitutively express *Il12a* [6]. In contrast, flagellin treatment induced many fewer lymphoid cells expressing the GREAT IFN-γ reporter and also did not induce increased amounts of *Il12b* mRNA.

To determine whether IL-12 was responsible for the production of IFN-γ, we injected mice with a neutralizing antibody against IL-12 p40, and then sensitized the mice i.n. with OVA plus CpG ODN as before. Treatment with anti-IL-12 p40 reduced substantially the innate IFN-γ production in response to CpG ODN (Fig 4D). Moreover, blocking IL-12 during the sensitization phase abrogated the adaptive T_H_1 response seen upon rechallenge with OVA in the mice sensitized with OVA plus CpG ODN (Fig 4E). In contrast, treatment of anti-IL-12 did not decrease either the percentage of CD8 T cells producing IFN-γ or the anti-OVA IgG2c response after antigen rechallenge (Fig 4F), suggesting these responses required lower levels of IL-12 [6] or they were promoted by other cytokines, such as type 1 IFNs [3].

To determine how IL-12 was produced in response to i.n. immunization with OVA plus CpG ODN, we used the *Myd88*^*fl/fl*^ *CD11c-Cre* mice. In these mice, MyD88, a signaling component that is required for signaling by most TLRs [40] and by receptors for IL-1 family cytokines [41], is deleted in almost all cDCs, ~80% of plasmacytoid DCs, and in some macrophage subsets, including AMs [4,42]. *Myd88*^*fl/fl*^ *CD11c-Cre* mice and control mice were i.n. treated with OVA plus CpG ODN, and after 2 h the levels of cytokine mRNAs in the lung were determined. Although the cytokine responses at this early time may result from direct TLR stimulation, we cannot rule out a rapid response to an IL-1 family cytokine. In response to CpG ODN, the mutant mice produced dramatically less *Il12b* mRNA compared to control mice (Fig 4G), indicating that cDCs and/or AMs produced IL-12 in response either to direct stimulation by CpG ODN via TLR9, or to indirect stimulation by an IL-1 family cytokine that was released very rapidly by another cell type. Consistent with these genetic data, cDC subsets and AMs sorted from the lung after OVA plus CpG ODN treatment expressed more *Il12b* mRNA than the corresponding cells sorted from the lung after OVA treatment (Fig 4H). Both major cDC subsets, CD103^+^ DCs and CD11b^+^ DCs, had higher levels of *Il12b* mRNA normalized to a housekeeping gene compared to AMs. cDC subsets and AMs also had induced *Il12a* after CpG ODN treatment (Fig 4I). CD103^+^ cDCs had a greater fold induction of *Il12a* and higher relative mRNA values compared to AMs and CD11b^+^ cDCs. In the *Myd88*^*fl/fl*^ *CD11c-Cre* mice, in which cDCs and AMs cannot respond to CpG ODN and failed to induce *Il12b* mRNA, the innate IFN-γ response and adaptive T_H_1 response were completely abrogated in response to CpG ODN (Fig 4J–4L). These results are in agreement with the experiments described above in which blocking IL-12 with an antibody inhibited IFN-γ responses.

### Flagellin, but not CpG ODN, promotes the appearance of IL-4-producing CD4 T cells and follicular helper T cells in the lung draining LN

**Fig 5:**
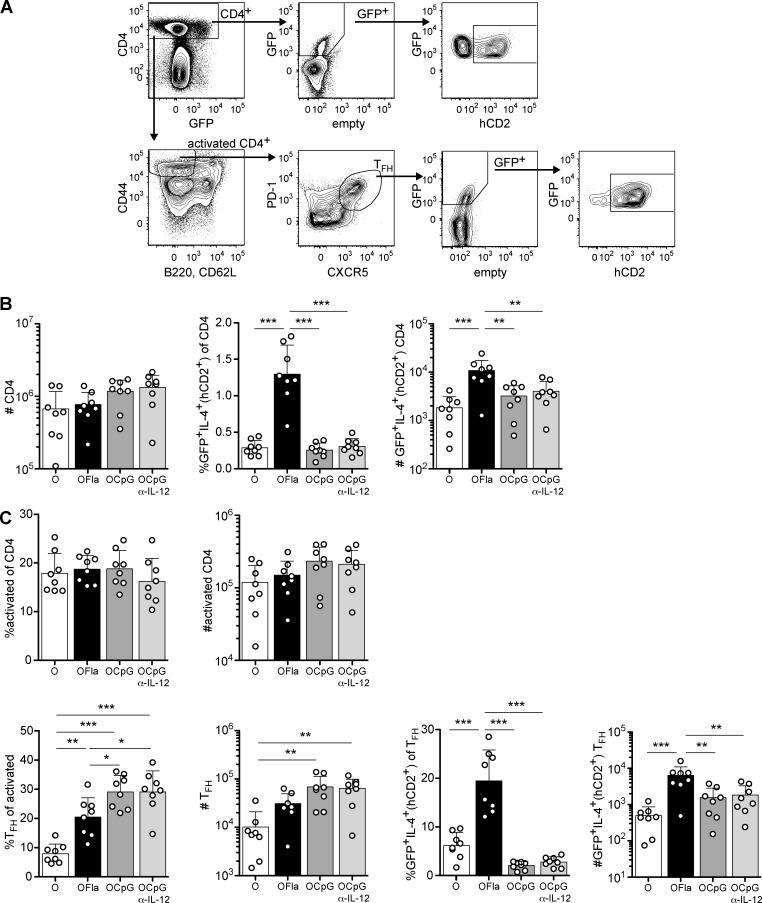
Flagellin, but not CpG ODN, promotes development of IL-4-producing CD4 T cells in the draining LN. 4get/KN2 reporter mice were administered OVA, OVA plus flagellin (1 μg), or OVA plus CpG (0.75 μg) i.n. on d0, 1 and 2. In addition, to block IL-12 action in some mice, mice were given anti-IL-12 p40 or control antibody (rat IgG2a) twice, one day before initial sensitization (700 μg i.p.) and again on d2 (300 μg i.p.). On d6, expression of IL-4 reporters (GFP^+^hCD2^+^) by CD4 T cells was examined in the mediastinal LN. (**A**) Gating strategy of CD4 T cells (CD4^+^), activated CD4 T cells (CD4^+^CD44^hi^B220^-^CD62L^-^), and T_FH_ cells (CD4^+^CD44^hi^B220^-^CD62L^-^PD-1^+^CXCR5^+^). (**B**) Numbers of CD4 T cells, percentages and numbers of GFP^+^IL-4^+^(hCD2^+^) CD4 T cells. (**C**) Percentages and numbers of activated CD4 T cells, percentages and numbers of T_FH_ cells, and percentages and numbers of GFP^+^IL-4^+^(hCD2^+^) T_FH_. Data are pooled from two independent experiments with combined totals of 8 mice per group. Each circle represents one individual mouse. Error bars indicate mean +SD. * P ≤ 0.05, ** P ≤ 0.01, *** P ≤ 0.001 using one-way anova with Bonferroni post-test.

To gain insight into the mechanism by which flagellin induced a T_H_2 response, we next examined IL-4-producing cells in the lung-draining mediastinal LN after i.n. immunization with OVA plus flagellin (Fig 5). Mice sensitized with OVA plus flagellin had a greater number of LN CD4 T cells that had secreted IL-4 based on hCD2 reporter expression than mice sensitized with OVA alone or with OVA plus CpG ODN (Fig 5B). Follicular helper T cells (T_FH_) are known to be important in the formation and maintenance of germinal centers, where B cells differentiate into long-lived plasma cells and affinity-matured memory B cells [43]. Addition of flagellin or CpG ODN as adjuvant increased the fraction of activated CD4 T cells that became T_FH_, as defined by the dual expression of PD-1 and CXCR5 (Fig 5C). Use of flagellin, but not CpG ODN, as adjuvant also strongly increased the fraction of T_FH_ that had secreted IL-4 as assessed with the KN2 reporter, correlating with the production of IgE anti-OVA antibodies (Fig 1C). These data are in agreement with the conclusion that i.n. immunization with flagellin as adjuvant leads to a predominant T_H_2 response in the lung.

### Flagellin induces innate production of cytokines associated with induction of T_H_2 responses

Some investigators have previously suggested that T_H_2 responses may be a default response that occurs when there is limited IL-12 and IFN-γ production during an inflammatory response [44,45]. Therefore, we examined IL-4 production by CD4 T cell in the draining LN of mice treated with anti-IL-12 p40 antibody and then sensitized with OVA plus CpG ODN. These mice did not exhibit IL-4 production in CD4 T cells on d6 in the draining LN, based on lack of expression of the KN2 reporter (Fig 5B and 5C). These results suggest that flagellin, but not CpG ODN, induced production of one or more cytokines that contributed positively to the induction of T_H_2 cells.

**Fig 6:**
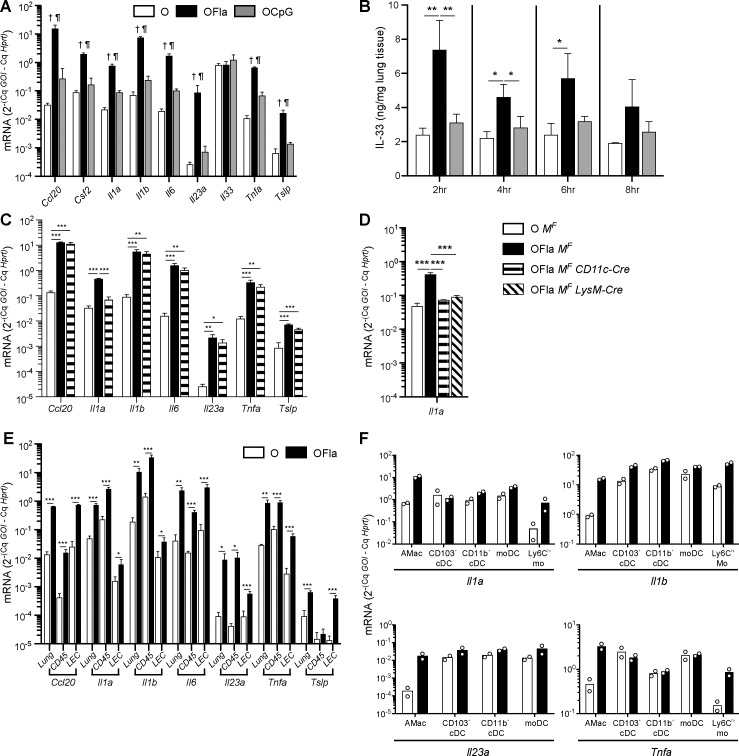
Flagellin induces rapid production of inflammatory cytokines by AMs, LECs, Ly6C^hi^ monocytes, and CD103^+^ cDCs. Inflammatory gene mRNA inductions and IL-33 protein in whole lung tissue (**A-E**), enriched lung cell populations (**E**), or sorted cell populations from the lung (**F**) after one i.n. administration of OVA, OVA plus flagellin (1 μg), or OVA plus CpG (3 μg). mRNA inductions were normalized to *Hprt*. (**A**) Inflammatory gene mRNA inductions in whole lung tissue 2h after i.n. administration. (**B**) IL-33 protein in whole lung homogenates at the indicated time points. (**C-F**) Inflammatory gene mRNA inductions were measured in whole lung tissue (**C**, **D**), in fractionated lung epithelial or hematopoietic-derived cell populations (**E**), or in sorted cell populations (**F**) 2h after i.n. administration of *Myd88*^*fl/fl*^ mice (*M*^*F*^), *Myd88*^*fl/fl*^ *CD11c-Cre* (*M*^*F*^ *CD11c-Cre*) mice, or *Myd88*^*fl/fl*^ *LysM-Cre* (*M*^*F*^ *LysM-Cre*) mice. Data in (**A**) contain 5 mice per group and are representative of two independent experiments, data in (**B**) contain 3 mice per group and are representative of two independent experiments at each time point, data in (**C**) contain 3–5 mice per group and are representative of three independent experiments, data in (**D**) contain 3 mice per group and are representative of two independent experiments, data in (**E**) contain 3–4 mice per group and are representative of two independent experiments, and data in (**F**) are pooled from two independent experiments with combined totals of 6–7 mice per group; each circle represents the data from sorted cells obtained from 3–4 mice. Error bars indicate mean +SD. In (**A**), statistical differences (P ≤ 0.05) are indicated with the following symbols: O vs. OFla (†), and OFla vs. OCpG (00B6).* P ≤ 0.05, ** P ≤ 0.01, *** P ≤ 0.001 using one-way anova with Bonferroni post-test (**A**-**D**) or Student’s *t*-test within the same tissue/cell population (**E**).

Therefore, we next examined the mRNA and protein expression of cytokines that have been implicated as being important for inducing type 2 immune responses in the lung [10–12]. Flagellin induced the mRNA that encodes TSLP (Fig 6A), and increased protein levels of the mature form of IL-33 (Fig 6B). IL-33 production appeared to be a post-transcriptional response, as there were only small changes in *Il33* mRNA (Fig 6A). In contrast, CpG ODN induced little or no *Tslp* mRNA or IL-33 release. IL-1α and IL-1β have also been implicated in promoting T_H_2 responses [13,14], and the mRNAs for these cytokines were also robustly induced by flagellin (Fig 6A).

We next used the *Myd88*^*fl/fl*^ *CD11c-Cre* mice to examine the extent to which MyD88 signaling by TLRs or IL-1 family receptors in cDCs and AMs contributed to the innate cytokine responses induced by flagellin. Deletion of MyD88 in these cell types largely unaffected flagellin’s ability to induce mRNAs that encode CCL20, IL-1β, TNFα, IL-6, IL-23 p19, and TSLP (Fig 6C), suggesting flagellin stimulated other cell types, such as LECs or a hematopoietic-derived non-AM, non-cDC population, to induce these cytokines. In contrast, these mutant mice exhibited a large decrease in the induction of *Il1a* mRNA (Fig 6C). To determine whether cDCs or AMs were responsible for the induction of *Il1a* mRNA, we used *Myd88*^*fl/fl*^ *LysM-Cre* mice, which delete *Myd88* in AMs with similar efficiency to the *Myd88*^*fl/fl*^ *CD11c-Cre* mice, but do not delete *Myd88* in DCs [42]. Both types of mutant mice exhibited a similar decrease in *Il1a* mRNA induction (Fig 6D). These results suggest that AMs may be the major cell contributor of *Il1a* mRNA in the lung.

To further investigate what cell types responded rapidly to flagellin and induced *Tslp* and the other inflammatory cytokine mRNAs, we fractionated the lung into two subpopulations, one enriched for LEC and the other enriched for hematopoietic-derived CD45^+^ cells 2 h after treatment with OVA or OVA plus flagellin (Fig 6E). *Ccl20* and *Tslp* mRNAs were induced after flagellin stimulation in the LEC fraction with similar magnitude as in whole lung tissue, whereas the CD45^+^ cell fraction had little to no induction of *Ccl20* and *Tslp* mRNA, indicating that LEC are the major producers of *Ccl20* and *Tslp* mRNAs. In contrast, several other inflammatory cytokines were induced in both cell populations. Flagellin treatment induced substantial levels of *Il6* mRNA in both LEC and CD45^+^ populations, with the LEC population expressing higher levels of this mRNA. *Il23a* and *Tnfa* mRNA was also induced in both the LEC and CD45^+^ cell fractions, but for these cytokines, CD45^+^ cells expressed higher levels of mRNA. Interestingly, *Il1a* and *Il1b* mRNA were induced only to a small degree in the LECs, and the CD45^+^ cell fraction was mainly responsible for the robust induction of these cytokines in the lung after flagellin treatment. Thus, although LECs appeared to be the major contributor of *Ccl20*, *Tslp*, and *Il6* mRNAs, CD45^+^ cells also responded rapidly to flagellin and contributed to early inflammatory cytokine production, especially for *Il1a*, *Il1b*, *Il23a*, and *Tnfa*.

To elucidate which CD45^+^ cell types may have been producing the latter cytokines, 2 h after OVA or OVA plus flagellin stimulation, we sorted AMs, CD103^+^ cDCs, CD11b^+^ cDCs, monocyte-derived DCs (moDCs), and Ly6C^hi^ monocytes from the lung, and measured their levels of mRNAs encoding these cytokines (Fig 6F). Consistent with the genetic evidence described above indicating that AMs may be the major producer of *Il1a* mRNA, AMs from flagellin-treated mice induced ~15 fold more *Il1a* mRNA than AMs from OVA-treated mice and had the highest level of *Il1a* mRNA normalized to a housekeeping gene compared to the other cells examined. AMs isolated after *in vivo* flagellin stimulation also exhibited strong inductions of *Il1b*, *Il23a* and *Tnfa* mRNAs. However, the induced levels of *Il1b* and *Il23a* mRNAs by AMs were not higher than the induced levels in some of the other cell types. In particular, the various DC subtypes examined all had greater than 2-fold higher levels of *Il1b* and *Il23a* mRNA than AMs and had ~2-3-fold higher levels from mice stimulated with OVA plus flagellin compared to mice stimulated with OVA alone. In addition, Ly6C^hi^ monocytes from flagellin-treated mice had ~5-fold greater induction of *Il1b* and *Tnfa* compared to these cells from OVA-treated mice.

### Migratory DCs require MyD88 signaling to respond normally to i.n. exposure to flagellin or CpG ODN

**Fig 7:**
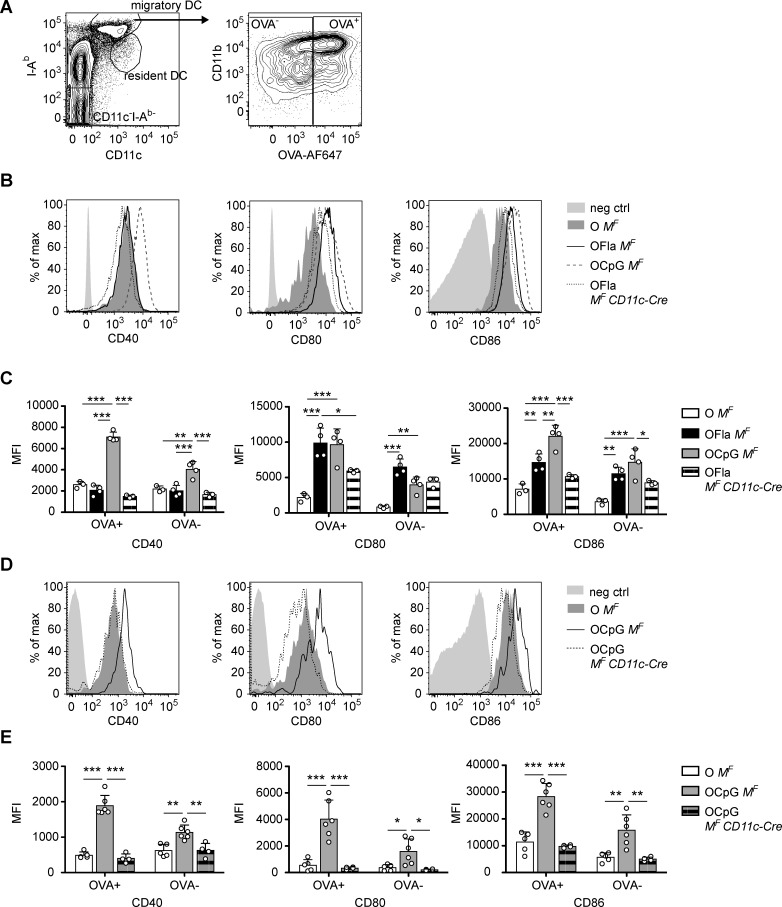
Migratory DCs require MyD88 signaling to respond normally to i.n. exposure to flagellin or CpG ODN. Expression of activation markers on migratory DCs in the lung-draining, mediastinal LNs of *Myd88*^*fl/fl*^ (*M*^*F*^) and *Myd88*^*fl/fl*^ *CD11c-Cre* (*M*^*F*^ *CD11c-Cre*) mice one day after i.n. administration (d1) of OVA-AF647 or OVA-AF647 plus TLR ligand. (**A**) Migratory DCs were gated as CD11c^+^I-A^b(hi)^, then gated according to OVA-AF647 expression. (**B**) and (**C**) Comparison of different activation markers on migratory DCs between *M*^*F*^ and *M*^*F*^ *CD11c-Cre* mice treated i.n with OVA-AF647, OVA-AF647 plus flagellin (1 μg), or OVA-AF647 plus CpG (0.75 or 3 μg). (**B**) Representative histograms of different activation markers on migratory DCs that did take up OVA-AF647. (**C**) Level of expression (MFI) of activation markers on migratory DCs that did (OVA^+^) or did not (OVA^-^) take up fluorescent OVA. (**D**) and (**E**) Comparison of different activation markers on migratory DCs between *M*^*F*^ and *M*^*F*^ *CD11c-Cre* treated i.n with OVA-AF647 or OVA-AF647 plus CpG ODN (0.75 μg). (**D**) Representative histograms of different activation markers on migratory DCs that did take up OVA-AF647. (**E**) Level of expression (MFI) of activation markers on migratory DCs that did (OVA^+^) or did not (OVA^-^) take up fluorescent OVA. Data in (**B**) and (**C**) contain 3–4 mice per group and are representative of 3 independent experiments using OVA-AF647 and a fourth independent experiment using non-fluorescent OVA. Data in (**D**) and (**E**) contain 4–6 mice per group and are representative of two independent experiments. Negative control histograms (solid light gray) were from CD11c^-^I-A^b-^ cells. Each circle represents an individual mouse. Error bars indicate mean +SD. * P ≤ 0.05, ** P ≤ 0.01, *** P ≤ 0.001 using one-way anova with Bonferroni post-test.

DCs are important for bridging innate and adaptive immune responses by their ability to take up antigens, to migrate from a tissue location to the draining LN, and to present antigens to T cells [3]. Therefore, we examined migratory DCs (CD11c^+^I-A^b(hi)^) in the mediastinal LN one day after i.n. administration of OVA with flagellin or CpG ODN (Fig 7A). OVA that was fluorescently labeled with Alexa Fluor 647 (OVA-AF647) was used to track the migratory DC that had taken up the antigen. Using flagellin or CpG ODN with OVA led to the upregulation of CD80 and CD86 on the surface of migratory DCs in the mediastinal LN that had taken up OVA-AF647, whereas using CpG ODN additionally led to the upregulation of CD40 (Fig 7B and 7C). The draining LN also contained migratory DCs that did not have any fluorescence and therefore were likely mobilized by responding to the cytokines produced in the lung. These migratory DCs lacking OVA also upregulated the co-stimulatory molecules, but to a lesser degree. Thus, both flagellin and CpG ODN led to DC activation, although the patterns of costimulatory molecule expression differed. In addition, treatment with flagellin or CpG ODN led to similar degrees of upregulation of CD40, CD80, and CD86 on the surfaces of migratory CD103^+^ cDCs and CD11b^+^ DCs subsets in the mediastinal LNs (S4 Fig).

To determine how TLRs and/or IL-1R family member signaling within DCs contributed to their activated phenotype, we again used the *Myd88*^*fl/fl*^ *CD11c-Cre* mice. These mice treated with CpG ODN had little to no upregulation of activation markers on their migratory DCs (Fig 7D and 7E), suggesting that TLR and/or IL-1R family signaling in DCs was necessary for their activation. In contrast, *Myd88*^*fl/fl*^ *CD11c-Cre* mice sensitized with OVA-AF647 plus flagellin, had a lesser, partial defect in CD80 upregulation in migratory DCs that had taken up antigen. Also, mutant migratory DCs showed a partial reduction in the expression of CD86, although this trend was not statistically significant (Fig 7B and 7C). Thus, in mice treated with flagellin, the induction of CD80 on migratory DCs from the lungs may be partially dependent on MyD88-dependent and MyD88-independent stimulation of the DCs.

### Multiple cytokines are likely involved in flagellin-induced T cell polarization

**Fig 8:**
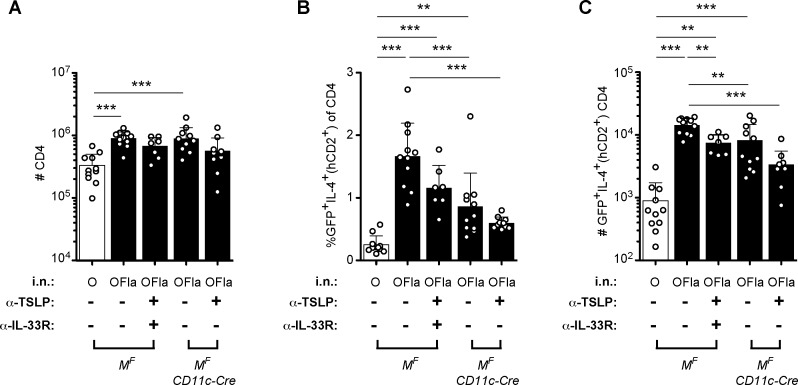
Multiple cytokines are likely involved in flagellin-induced T cell responses. *Myd88*^*fl/fl*^ (*M*^*F*^) and *Myd88*^*fl/fl*^ *CD11c-Cre* expressing the 4get/KN2 reporter were treated with anti-TSLP (IgG2a), anti-IL-33R (IgG1), and/or appropriate control antibodies (rat IgG2a, rat IgG1), one day before initial sensitization (i.p. 250 μg anti-TSLP, 160 μg anti-IL-33R, or corresponding amounts of appropriate isotype controls), and anti-IL-33R or rat IgG1 again on d2 (i.p. 160 μg). These mice were then administered i.n. OVA or OVA plus flagellin (1 μg) on d0, 1, and 2. On d6, expression of IL-4 (GFP^+^hCD2^+^) by CD4 T cells in the mediastinal LN was examined using the same gating strategy as in Fig 5. (**A**) Numbers of CD4 T cells, (**B**) percentages and (**C**) numbers of GFP^+^IL-4^+^(hCD2^+^) CD4 T cells. Data are pooled from three independent experiments, one of which did not have the anti-TSLP and IL-33R treatment group, with combined totals of 7–11 mice per group. The comparisons between *M*^*F*^ mice treated i.n. with OVA or OVA plus flagellin, and *M*^*F*^ *CD11c-Cre* mice treated i.n. with OVA plus flagellin are representative of three additional independent experiments. Each circle represents one individual mouse. Error bars indicate mean +SD. * P ≤ 0.05, ** P ≤ 0.01, *** P ≤ 0.001 using one-way anova with Bonferroni post-test.

Because i.n. sensitization with OVA plus flagellin led to IL-33 production and induction of mRNA encoding TSLP, two cytokines implicated in promoting type 2 immune responses, we tested the requirement for these two cytokines by neutralizing TSLP with anti-TSLP [46] and/or blocking IL-33R signaling with an antibody against the ST2 subunit of the IL-33R. Mice that were immunized with OVA plus flagellin after blocking TSLP and IL-33R still exhibited a statistically significant increase in the fraction of CD4 T cells producing IL-4 on d6 in the draining LN, compared to these cells in control mice immunized with OVA alone (Fig 8). Blocking TSLP and IL-33R may have been reduced the fraction of IL-4-producing CD4 T cells, but this difference was not statistically significant. These results suggested that additional cytokines may have also contributed to the IL-4 response in T cells. As described previously, flagellin also induced the mRNA encoding IL-1α and IL-1β, cytokines that have also been suggested to be involved in T_H_2 polarization in some circumstances [13,14]. To address this possibility, we examined the d6 CD4 T cell response in the *Myd88*^*fl/fl*^ *CD11c-Cre* mice, which are unable signal through TLR, IL-1R or IL-33R in cDCs and AMs. When these mice were immunized with OVA plus flagellin, the fraction of CD4 T cells that had secreted IL-4 was intermediate between the control mice treated with OVA plus flagellin and the control mice that were treated with OVA alone (Fig 8). To see if TSLP contributed to the residual response in *Myd88*^*fl/fl*^ *CD11c-Cre* mice, we treated these mice with anti-TSLP before i.n. sensitization with flagellin plus OVA. The percentage of IL-4-producing CD4 T cells in the draining LN was now clearly less than the control-treated mice immunized with OVA plus flagellin, and the percentage was not statistically greater than the response of control mice immunized with OVA alone (Fig 8). Thus, the early IL-4 production in CD4 T cells in the draining LN induced by flagellin may be partly due to MyD88-dependent stimulation of DCs. Furthermore, this response may also be partly due to MyD88-independent stimulation of the DCs, which at least partly involved TSLP acting on DCs and/or other cell types.

## Discussion

To gain insights into the development of allergic immune responses in the lung, we adapted a previous model of repeated airway immunization that used OVA and low dose LPS as adjuvant [21,22] to test the effects of other TLR ligands on the resulting adaptive immune response. Remarkably, we found that two TLR ligands, which each signal primarily via the adaptor molecule MyD88, both enhanced the adaptive immune response to OVA, but induced different polarized responses. CpG ODN, a TLR9 ligand, induced a T_H_1 response and IgG2c anti-OVA antibodies, whereas flagellin, a TLR5 ligand, induced a T_H_2 response and IgE anti-OVA antibodies. In addition, we characterized the key cells and cytokines that promoted T_H_1 versus T_H_2 responses in the lung using these different adjuvants. CpG ODN administered via the airways most likely was recognized directly by TLR9 on cDCs and AMs, leading to early IL-12 production. IL-12 was necessary for the subsequent IFN-γ production by a variety of innate and adaptive lymphocytes. Conversely, flagellin apparently stimulated LECs and several types of hematopoietic cells to produce multiple cytokines implicated in T_H_2 polarization, including TSLP, IL-33, IL-1α, and IL-1β. These cytokines likely acted in an overlapping fashion to induce a T_H_2 response.

Mice treated with OVA plus CpG ODN induced a high level of IL-12, whereas mice treated with OVA plus flagellin induced much less IL-12. This difference may have facilitated the robust T_H_2 response that developed in the latter case, since a developing T_H_1 response can inhibit the generation of a T_H_2 response [7]. However, mice pre-treated with neutralizing antibody to IL-12 p40 and then sensitized with CpG ODN plus OVA did not have increased numbers of IL-4-producing CD4 T cells on d6 in the draining LN. These results suggested that flagellin acted positively to induce a T_H_2 response to OVA. Indeed, flagellin stimulated the production of a diverse array of cytokines previously implicated in T_H_2 polarization, including TSLP, IL-33, IL-1α and IL-1β [9,13,14,47]. In contrast, CpG ODN induced much lower levels of these cytokines. In agreement with our observations, a previous study also detected IL-33 and TSLP in the lung airspace after flagellin exposure [20], and other studies have found mRNA encoding IL-1α in lung after flagellin treatment [23,35].

When we further analyzed the inflammatory cytokines induced 2 h after i.n. treatment with flagellin, we found LECs had induced mRNAs encoding CCL20, IL-6, TSLP, and to a lesser extent, TNFα. These results suggest that LECs directly responded to flagellin *in vivo*, as indicated by previous experiments that showed TLR5 expression on non-hematopoietic cells played an important role in the innate [23,35,48] and adaptive [20,23] immune responses to flagellin in the lung. Moreover, a previous study showed that isolated LECs stimulated *in vitro* with flagellin produced TSLP [20]. Thus, our results join the emerging view that LECs play an active role in promoting inflammation and immune responses in the lung from their position at the barrier with the environment [14].

CD45^+^ immune cells in the lung also contributed significantly to the early production of many of the induced inflammatory cytokines. Two hours after flagellin stimulation, the CD45^+^ cells in the lung had higher expression than LECs of the mRNAs encoding IL-1α, IL-1β, IL-23 p35, and TNFα, whereas the CD45^+^ cells apparently had less *Il6* mRNA than LECs and almost none of the induced *Tslp or Ccl20* mRNA. AMs, Ly6C^hi^ monocytes, and DCs were substantial contributors to this cytokine induction, as assessed by sorting these cell types from i.n. immunized mice and measuring their mRNA levels. AMs from flagellin-treated mice induced the highest mRNA levels of *Il1a*, whereas the major DC subsets (CD103^+^ cDCs, CD11b^+^ cDCs and monocyte-derived DCs) expressed substantial amounts of mRNA encoding IL-1β and IL-23 p35. Ly6C^hi^ monocytes expressed similar levels of *Il1b* mRNA as the DC subsets. The effects of deleting the gene encoding the signaling component MyD88 in AMs also indicated that these cells were the major producer of IL-1α mRNA, but that other cells contributed more substantially to the production of other inflammatory cytokines. Because flagellin led to the rapid release of IL-33 and induction of IL-1 mRNA, it is not clear from our studies whether the cytokines induced by AMs or DCs were due to direct TLR5 signaling and/or IL-1R/IL-33R signaling. Supporting the idea that CD45^+^ can be directly stimulated by flagellin, a previous study showed that AMs can produce cytokines when stimulated with flagellin *in vitro* [49], and other studies have shown that TLR5 expression in hematopoietic cells was necessary for some of the cytokine production seen in response to i.n. exposure to flagellin [35,48]. However, another study showed TLR5 expression on non-hematopoietic cells, but not hematopoietic cells, was necessary for induction of the cytokines they examined [23]. The discrepancies between this study and others [35,48] may be because the analyses varied in terms of time points and cytokines examined, and whether cytokine induction or production in hematopoietic cells were directly analyzed.

We found that i.n. treatment of OVA plus CpG ODN led to rapid induction of the mRNA encoding for IL-12 p40, but this induction was completely absent in similarly treated *Myd88*^*fl/fl*^ *CD11c-Cre* mice, indicating that MyD88 signaling in AMs and/or DCs was required for the response. Furthermore, we found AMs and cDCs sorted from mice treated with CpG ODN for 2 h induced both *Il12a* and *Il12b* mRNA. Previous studies have reported that lung DCs stimulated *in vitro* with TLR9 ligands produced IL-12 p40 [24,50], and that lung DCs can produce IL-12 p70 [25]. Although, there are conflicting reports as to whether or not AMs express TLR9 and are capable of responding to CpG ODN *in vitro* [23,25,50], our results support the idea that AMs and DCs can respond directly to CpG ODN *in vivo*.

The mechanisms by which DCs bridged innate and adaptive immune responses were somewhat different in response to OVA plus CpG ODN versus OVA plus flagellin. DC activation after i.n. OVA plus CpG ODN likely required direct TLR9/MyD88 signaling in the DCs as induction of *Il12b* mRNA, activation of migratory DCs, and induction of innate and adaptive IFN-γ production were completely abrogated in *Myd88*^*fl/fl*^ *CD11c-Cre* mice. In contrast, these mutant mice exhibited a partial, rather than complete, defect in expression of activation markers on DCs after i.n. OVA plus flagellin. This observation suggests that DC maturation after flagellin treatment resulted from a combination of signaling through receptors that require MyD88 and those that do not. In agreement with this conclusion, sensitization with flagellin induced mRNAs encoding MyD88-dependent cytokines such as IL-1α and IL-1β, and led to the release of IL-1 family member, IL-33. Flagellin also induced mRNAs encoding for cytokines such as IL-6, TNFα, and TSLP that act via receptors that do not require MyD88.

As discussed above, our study did not directly address whether lung DCs responded directly to flagellin via TLR5 or whether the MyD88-dependent component of their response was due to IL-1α, IL-1β, and/or IL-33. A recent study using mixed bone-marrow chimeras found that DC maturation in response to i.n. flagellin administration can occur in the absence of TLR5 expression in DCs [36], suggesting that cytokines produced by other cell types, rather than direct TLR signaling via MyD88 in DCs, may be activating DCs in response to i.n. flagellin. However, another study showed TLR5 expression on hematopoietic cells were important for some aspects of the adaptive immune response induced by flagellin [20]. Thus, it unclear which hematopoietic cell type (DCs, AMs, or another cell type) is required to directly respond to flagellin to promote some aspects of the adaptive immune response.

A previous study showed that CD11b^+^ cDCs, and not CD103^+^ cDCs, were important for flagellin-induced CD4 T response *in vitro* and in an adoptive-transfer model [36]. In other model systems, namely the house dust mite model, there are conflicting data on which DC subset is the most important [51–53]. In our studies, both CD103^+^ and CD11b^+^ DCs from flagellin-treated mice upregulated CD80 and CD86 more than occurred in those cells from OVA-treated mice. Furthermore, we did not find any major differences in cytokine induction in these cell populations, though we only examined a small set of genes, and at one early time point. Thus, further studies should be performed using mice deficient in Klf4 [52], a transcription factor necessary for the development of CD11b^+^ cDCs, to test which lung DC subset is necessary *in vivo* for the induction of the T_H_2 response when flagellin is used as an adjuvant.

Previous studies have shown that TSLP and IL-33 can individually promote T_H_2 polarization in some circumstances [9,47]. Moreover, in some model systems, these cytokines are necessary for T_H_2 induction [9,47]. However, IL-1α and IL-1β have also been implicated in promoting T_H_2 responses [13,14]. When we treated mice with blocking antibodies against TSLP and IL-33R prior to i.n. administration of OVA plus flagellin, the fraction of CD4 T cells producing IL-4 in the draining LN was not significantly reduced from control mice. This result suggests that these two cytokines alone or in combination were not wholly responsible for T_H_2 polarization in this model. In contrast, IL-4 production by CD4 T cells on d6 following sensitization with OVA plus flagellin was partially blocked in *Myd88*^*fl/fl*^ *CD11c-Cre* mice, which are defective in signaling via TLRs and receptors for IL-1 and IL-33. When these mice were additionally treated with anti-TSLP, the IL-4 production by CD4 T cells on d6 was indistinguishable from that of mice treated with OVA alone. A straightforward interpretation of these results is that IL-1 and/or IL-33 signaling directly in cDCs and/or in AMs, together with TSLP signaling in these cells and/or others, contributed importantly to the T_H_2 response. Alternatively, it is also possible that TLR5 signaling in cDCs and/or AMs contributed, along with TSLP and possibly other cytokines. The multiple pathways required for flagellin-induced IL-4 CD4 T cell production were in stark contrast to the more direct mechanism of T_H_1 induction by CpG ODN. Neutralizing IL-12 was sufficient to block innate IFN-γ production and the subsequent T_H_1 response following sensitization with OVA plus CpG ODN. In addition, direct sensing of CpG ODN by cDCs and/or AMs was required for IL-12 production by these cell types, and the subsequent innate and adaptive IFN-γ production.

Several previous studies have investigated the adjuvant activity of flagellin when administered into the lungs [20,23,36]. Although our and other studies differed somewhat in the source and endotoxin-level of flagellin, the sensitization and challenge regime, and the experimental read-outs, all our results support that global conclusion that flagellin as an adjuvant promotes a robust T_H_2 responses in the lungs in B6 mice. Interestingly, a previous study found when flagellin was given in the airways of BALB/c mice after sensitization by OVA/alum or by house dust mite, flagellin inhibited the developing T_H_2 response [54]. Furthermore, bone-marrow-derived DCs stimulated with an OVA-flagellin fusion protein produced IL-10 [55], and these stimulated DCs could inhibit T_H_2 cytokine production *in vitro* [56]. These studies suggest the immunological context in which flagellin is administered is critical in determining flagellin’s effect on the subsequent immune response.

Given the complexity we found in the mechanism by which flagellin conditioned DCs to promote the type 2 polarized response of draining lymph node CD4 T cells, it is perhaps not surprising that there are some results that do not fully agree between our studies and some of the previous studies. One potentially significant methodological difference is that in our studies, we used cytokine reporter mice to determine whether lung-infiltrating CD4 T cells made IL-4, IFN-γ or IL-17A *in vivo*, rather than using *in vitro* restimulation with antigen. While each approach has its strengths, cytokine reporter mice allowed us identify other types of immune cells in the lung that were making the cytokines of interest. For example, we observed that on d22 there was substantial IL-4 production by basophils, which may have responded via OVA-specific IgE bound to their FcεRI [29]. It is likely that the CD4 T cells made these cytokines largely in response to antigenic stimulation *in vivo*, but for other cell types, cytokine production may be responsive to other inputs, such as IL-12 promoting IFN-γ production by NK cells. In any case, the character of the immune responses seen after using CpG ODN or flagellin were consistent with regard to polarization of CD4 T cells, the isotype of anti-OVA antibodies produced, and the character of the induced inflammation seen in the airways during the challenge phase. Moreover, the data from the cytokine reporter mice were in general agreement with previous studies using *in vitro* restimulation of mediastinal LN cells with antigen after similar sensitization [20,23].

While the effector CD4 T cell response in our system was dominated by T_H_2 effectors, as indicated both by cytokine reporter expression and by the character of the inflammatory infiltrate during the challenge phase of the response, there may have been a lesser component of T_H_1 or T_H_17 cells. For example, we found 3–4 fold increases in IFN-γ-producing CD4 T cells and IL-17-producing CD4 T cells in the lungs of flagellin-treated mice compared to OVA-treated mice, but these increases were not statistically significant. Van Maele et. al.[23] and Wilson et. al.[20], using somewhat different sensitization regimes, did observe statistically significant increases of IFN-γ and IL-17, respectively, from lymph node and/or lung cells cultured with OVA, suggesting a partially mixed T_H_1-T_H_2-T_H_17 response in those systems. Also, Fougeron et. al. [36] found that CD11b^+^ cDCs produced IL-12 p40 18 h after flagellin treatment, which suggests that a partial T_H_1 response may have been induced.

Extracts of allergenic organisms typically contain multiple classes of allergens that stimulate the immune system through different pathways, so dissecting how they induce T_H_2 responses is highly challenging. Therefore, we developed a reductionist system to understand how one component that is present in allergenic extracts and signals through a well characterized innate immune pathway, namely the TLR5 ligand, flagellin, can promote T_H_2 responses in the lung. We based the sensitization and rechallenge regime on previous studies showing that a low dose of a TLR4 ligand, LPS, as an i.n. adjuvant led to a robust T_H_2 response and eosinophil infiltration into the lung, characteristics of acute asthma, whereas, a high dose of LPS led to a T_H_1 response and infiltration of neutrophils into the lung upon subsequent challenge with antigen [21,22]. However, as the high dose of LPS induced a more robust innate inflammatory response than the lower dose of LPS [57], the magnitude of initial inflammation may be an important determinant of the T_H_1 and T_H_2 polarization in the LPS model. In contrast, flagellin induced a robust T_H_2 response over a wide dose response range, whereas CpG ODN induced a T_H_1 response at all doses tested (data not shown). Moreover, the doses of flagellin and CpG ODN used in these experiments induced similar magnitudes of innate inflammatory cell infiltrates on d3. Therefore, the model immunization developed in these studies provides an opportunity to dissect robust qualitative differences between two adjuvants acting via TLRs that signal via the adaptor MyD88, one that promoted a lung T_H_2 response and another that promoted a lung T_H_1 response.

Although asthma is predominantly understood as a dysfunctional T_H_2 response in the lung, recent studies have shown that some asthmatic patients have mixed T_H_2 and T_H_17 responses, or even predominant T_H_17 responses in the lung [58]. In our model of immunization, although neutrophils were prominent inflammatory cells and substantial percentages of γδ T cells were producing IL-17 during the innate phase of the response, a robust T_H_17 adaptive immune response was not observed in response to i.n. sensitization and challenge when flagellin or CpG ODN was used as adjuvant. Correspondingly, the inflammation following OVA rechallenge was dominated by eosinophils in the case of flagellin or by monocytes in the case of CpG ODN, consistent with T_H_2 and T_H_1 dominated responses, respectively.

Although clinically relevant allergens may stimulate the immune system through both TLR and other innate pathways, nonetheless, our results with a reductionist system may be relevant to the development of human asthma in many cases, as Wilson et. al. [20] found that four out of seven house dust extract samples collected from households in North Carolina contained flagellin. Mice sensitized and rechallenged with three of these flagellin-containing samples had eosinophil infiltration in the lung airspace after rechallenge, and this response was TLR5 dependent. House dust extract contains multiple allergens [8,59], so the adjuvant property of flagellin described here may be a significant contributor to development of asthma in some individuals.

## Materials and Methods

### Mice

Mice were used between the ages of 8 and 20 weeks. B6 mice (C57BL/6J or C57BL/6NCr) were purchased from Jackson Laboratory (Bar Harbor, ME) and National Cancer Institute (Frederick, MD), respectively. C57BL/6J were bred in the laboratory’s colony. *Myd88*^*fl/fl*^ mice were crossed to *CD11c-Cre*, as previously described [4], and *LysM-Cre* [60]. *Myd88*^*fl/fl*^ and *Myd88*^*fl/fl*^ *CD11c-Cre* mice were crossed to the following reporter mice to obtain mice with genotypes as described in the Figure Legends: GREAT–*Ifng*^*GREAT/GREAT*^ [33], 4get–*Il4*^*4get/4get*^ [30], KN2 –*Il4*^*KN2/KN2*^ [32], and SMART-17A –*Il17a*^*Smart/Smart*^ [34]. For Fig 2A–2C, two of three experiments used *Myd88*^*fl/+*^ *Il4*^*4get/KN2*^ *Mcpt8*^*Basopho8/+*^ mice [61], which gave similar results to one experiment using *Myd88*^*fl/fl*^ *Il4*^*4get/KN2*^ mice. *Tlr5*^*-/-*^ mice [35] were crossed to *Tlr11*^*-/-*^ [62] to generate *Tlr5*^*-/-*^*Tlr11*^*-/-*^. *Tlr4*^*-/-*^ mice were obtained from Jackson Laboratory. *Nlrc4*^*-/-*^ mice were obtained from Vishva Dixit (Genentech, Inc.). All mice had been backcrossed for at least 8 generations to C57BL/6J. Wildtype/control mice used included B6 mice and may have contained 1 or 2 alleles of the *Myd88*^*fl*^ allele and/or reporter alleles described above. Previous experiments have indicated that the *Myd88*^*fl*^ allele provides normal MyD88 function [4]. Mice were euthanized by administering anesthetizing dose of 2.5% 2,2,2-tribromoethyl alcohol solution (Sigma Aldrich, St. Louis, Mo), followed by bilateral thoracotomy. All mice were maintained in specific-pathogen free conditions, and used following UCSF Institutional Animal Care and Use Committee and NIH animal guidelines. The protocol (AN101733) used was approved by UCSF Institutional Animal Care and Use Committee.

### Reagents

Ovalbumin (OVA) Grade VI was purchased from Sigma Aldrich. Using a protocol from Aida and Pabst [63], Triton X-114 (EMD Millipore, Billerica, MA) was used to remove endotoxin from OVA. OVA depleted of endotoxin using this protocol contained <0.04EU/mg protein, as assayed using the *Limulus* amebocyte lysate (Lonza, Walkersville, MD). Endotoxin-depleted OVA was labeled with Alexa Fluor 647 using Alexa Fluor 647 carboxylic acid, succinimidyl ester (Life Technologies, Grand Island, NY). Phosphorothioate-backbone-containing CpG ODN 1826 (TCCATGACGTTCCTGACGTT), was purchased from Integrated DNA Technologies (Coralville, IA).

### Flagellin preparation

Flagellin was purified from *Salmonella typhimurium* TH4778, provided by K. Hughes (University of Utah), using a modified protocol from Smith et al. [64]. Briefly, highly motile bacteria growing exponentially were collected by centrifugation, suspended in 10 mM Tris buffer (pH 8.0), and blended in a blender (Waring Commercial, Stamford, CT) for 2 min to shear off flagella. After centrifugation for 15 min at 8,000xg to remove the bacteria, the supernatant fraction was serially ultracentrifuged twice for 1.25 h at 105,000xg to pellet the flagella, which were resuspended in Dulbecco’s (D)-PBS overnight at 4°C. This suspension was then heat depolymerized for 25 min at 70°C to dissociate flagellin polymers into monomeric flagellin subunits. The solution was then passed through an Amicon 100kDa MW cut-off filter (EMD Millipore, Billerica, MA) to remove polymers. The flagellin preparation was passed through an endotoxin removal column (EndoTrap; Hyglos, Charleston, SC) according to the manufacturer’s instructions. Protein concentration was quantified using the BCA Protein Assay Kit (Life Technologies). The preparation contained <0.013 EU/μg protein, as assayed using the *Limulus* amebocyte lysate.

### *In vivo* treatment

For i.n. administration, mice were briefly anesthetized with isofluorane and i.n. sensitized with endotoxin-depleted OVA (100 μg), OVA plus flagellin, or OVA plus CpG ODN in a total volume of 50 μl in D-PBS. The amounts of flagellin and CpG ODN used are specified in the Figure Legends. For mice rechallenged i.n. with OVA, 25 μg of OVA was used. For detecting reporter positive CD4 T cells, the time points examined after sensitization and rechallenge were chosen based on the most optimal time point (data not shown). In some experiments, mice were either treated intraperitoneally (i.p.) with neutralizing/blocking antibodies or with the appropriate rat isotype control(s). Anti-IL-12 p40 (C17.8) and rat isotype control IgG2a (2A3) were purchased from the UCSF Monoclonal Antibody Core (San Francisco, CA). Anti-IL-33Rα (IL1RL1, ST2) (DIH9) was purchased from BioLegend (San Diego, CA). The hybridoma producing anti-TSLP antibody (28F12) [46] was obtained from the Developmental Studies Hybridoma Bank, created by the NICHD and maintained at the Department of Biology, at the University of Iowa (Iowa City, IA). Bio X Cell (West Lebanon, NH) cultured the hybridoma and purified the resultant anti-TSLP antibody. Rat isotype control IgG1 (HPRN) and IgG2a (2A3) used in the anti-IL-33Rα, anti-TSLP experiments were purchased from Bio X Cell.

### Airway hyperresponsiveness (AHR) and mucus production

AHR and lung section preparation were performed on d21 as previously described [65]. Semi-quantitative scores were given to Periodic-acid Schiff-stained sections for mucus production by an observer blinded to the source of the samples as follows: grade 0 –none; grade 1 –<25% of airway epithelial cells; grade 2–25–50%; grade 3–51–75%; and grade 4 –>75%.

### Serum OVA-specific antibody quantification

High-binding polystyrene 96 half-well plates (Corning 3690, Sigma-Aldrich), were coated with OVA in D-PBS at 4°C overnight, blocked with 1% BSA, washed, and incubated with a dilution series of serum samples. The plate was then washed, incubated with anti-IgG2c-HRP (Southern Biotech, Birmingham, AL) or anti-IgE-biotin (BD Biosciences, San Jose, CA), followed by washing, and if necessary, incubated with streptavidin-HRP (Southern Biotech). The plates were then washed, incubated with 3,3',5,5'-Tetramethylbenzidine (Vector Labs, Burlingame, CA or KPL, Gaithersburg, MD) and read on a VERSAmax Microplate Reader (Molecular Devices, Sunnyvale, CA) at OD 450 nm and 570 nm. Relative titers were calculated by first plotting absorbance (A450-A570) versus antibody dilutions, and then calculating the antibody dilution (titer) at an absorbance within the linear range. Relative titers were normalized by comparison to a standard pooled serum from multiple experiments. Pooled serum from mice that were sensitized with i.n. flagellin plus OVA and then rechallenged with OVA was used for the anti-OVA IgE standard. Pooled serum from mice that were sensitized with i.n. CpG ODN plus OVA and then rechallenged with OVA was used for the anti-OVA IgG2c standard. In the cases where there was no or very low levels of a particular isotype detected in the least diluted serum samples, a limit of detection value was estimated using the slope from the standard and absorbance at the lowest dilution.

### Bronchoalveolar lavage (BAL) fluid and preparation of lungs and LNs into single cell suspensions for flow cytometry analysis and cell sorting

To collect BAL fluid, lungs were lavaged serially 3 times each with 1 ml D-PBS (Fig 1B) or with 1 ml HBSS with 5 mM EDTA (Fig 3A and 3B). To isolate lung cells, lungs were first perfused by cardiac puncture using 10 ml D-PBS, then excised and placed in a digestion solution containing Liberase TM (0.15–0.3 U/ml) (Roche, Indianapolis, IN) and DNase (40–50 U/mL) (Sigma Aldrich or Worthington, Lakewood, NJ) in HBSS with Ca^2+^ and Mg^2+^, supplemented with Penicillin-Streptomycin and 10 mM HEPES (pH 7.4) in a total volume of 5 ml. Lungs were either minced with scissors or dissociated with a tissue dissociator (Miltenyi Biotec, Auburn, CA), using program “lung_01”, and then incubated for 30 min at 37°C. For lungs minced by scissors, the lung suspensions were dispersed halfway during the incubation by pipetting up and down using a serological pipette. To stop digestion, EDTA and FBS were added. If using the tissue dissociator, samples were further dissociated using “lung_02”. For lungs minced by scissors, the lung suspensions were further dispersed by pipetting up and down using a serological pipette for 1 min. The lung suspension and any remaining lung tissue chunks were pushed through a 70 μm filter (BD Falcon, San Jose, CA). After centrifugation, the cells were resuspended in staining buffer (D-PBS/2% FBS/2 mM EDTA/0.1% sodium azide). Whole cell counts were obtained using the NucleoCounter (Chemometec, Davis, CA).

For cell sorting, lung cell suspensions were pooled from mice of the same treatment group, underlaid with 18% (w/v) Nycoprep solution (Accurate Chemical and Scientific Corporation, Westbury, NJ) and centrifuged at room temperature, at 450xg for 20 min to enrich for macrophages, DCs, and monocytes at the interface [66]. Cell suspensions were fractionated into CD11c^+^ and CD11c^-^ cells using anti-CD11c magnetic beads and LS columns (Miltenyi Biotec) according to the manufacturer’s instructions.

For assessing migratory DC phenotypes, mediastinal LNs were dissected and teased open and placed in tubes with 1 ml solution containing Liberase TM and DNase as described above. Digested LNs and LNs directly taken out of mice without digestion (Figs 5 and 8) were pushed through a 70 μm filter.

Lung and LN cells resuspended in staining buffer were incubated with anti-CD16/32 (2.4G2; UCSF Monoclonal Antibody Core), and then stained with a combination of antibodies for 30 min at 4°C. For the cell sorting experiments, the CD11c^+^ fraction was stained for markers that allow sorting for AMs, CD103^+^ cDCs, CD11b^+^ cDCs, and monocyte-derived DCs (moDCs). The CD11c^-^ fraction was stained for markers that allowed for sorting of Ly6C^hi^ monocytes. The following antibodies were from BD Biosciences: B220-APC-Cy7 (RA3-6B2); CD11c-biotin, -BUV395 (HL3); CD3-biotin (145-2C11); CD4-PE-Cy7 (RMA4-4); CD19-PE (1D3); CD40-PE (3/23); CD80-PE (16-10A1); CD86-PE (GL1); CXCR5-biotin (2G8); Ly6G-APC, -FITC (1A8); NK1.1-biotin, -PE, -PE-Cy7 (PK136); and SiglecF-PE (E50-2440). The following antibodies were from BioLegend: CD11b-PE-Cy7 (M1/70); CD11c-APC, -PerCp-Cy5.5 (N418); CD4-APC-Cy7, -PerCp-Cy5.5 (GK1.5); CD44-APC (IM7); CD49b-PerCp-Cy5.5 (DX5); CD62L-APC-Cy7 (MEL-14); CD64-PerCp-Cy5.5 (X54-5/7.1); CD8-PerCp-Cy5.5 (53–6.7); γ/δ TCR-APC, -FITC (GL3); CD103-FITC (2E7); human NGFR-PE (ME20.4); I-A^b^-PerCp-Cy5.5 (AF6-120.1); MHCII (I-A/I-E)-Brilliant Violet 605 (M5/114.152); Ly6C-APC-Cy7 (HK1.4); NK1.1-APC-Cy7 (PK136); and PD-1-PE-Cy7 (RMP1-14). The following antibodies were from eBiosciences (San Diego, CA): CD49b-biotin (DX5), Ly6C-APC-e780, -PerCp-Cy5.5 (HK1.4). The following antibodies were from Life Technologies: Mouse anti-human CD2-PE, and mouse IgG2a-PE. The following antibodies were from Tonbo Biosciences (San Diego, CA): B220-violetFluor 450 (RA3-6B2); CD3-violetFluor 450 (17A2); Ly6G-APC-Cy7 (1A8). The following antibodies were either from BD Biosciences or BioLegend: B220-APC-Cy7 (RA3-6B2); and Ly6G-PE (1A8). The following antibody was from either BioLegend or Tonbo Biosciences: CD3-PE-Cy7 (145-2C11). The tetramer for mCD1d-PE and -APC (PBSH7) was obtained from the NIH Tetramer Core Facility (Atlanta, GA). Biotinylated antibodies were labeled by incubation with streptavidin-Qdot605 (Life Technologies). For samples stained for CXCR5, after incubation with CD16/32, cells were incubated with anti-CXCR5-biotin at room temperature for 45–60 min. After washing, the cells were then were incubated with fluorophore-conjugated antibodies and streptavidin-Qdot605 for 30 min at 4°C. Prior to flow cytometry analysis, the cells were incubated with DAPI (Life Technologies) to facilitate exclusion of dead cells. Flow cytometry analysis was performed using a LSRII (BD Biosciences). Cell sorting was performed on a FACSAriaIII (BD Biosciences).

### Defining cell populations for flow cytometry analysis and cell sorting

Data were analyzed using FlowJo software (TreeStar, Ashland, OR) and cell sorting gates defined using FACSDiva 8 (BD Biosciences). For analysis and/or cell sorting of lung and BAL fluid cell suspensions, DAPI^-^ and DAPI^int^ cells were gated as “live”, because AMs are autofluorescent, and this DAPI^-/int^ gate was used to extrapolate total live cells (S5 Fig). In subsequent gating of lung and BAL cells, and for gating LN cells, other cell types were then identified as “live” based on lack of staining with DAPI.

Gates for cytokine reporter positive cells were based on cells from treated non-reporter control mice. In experiments using the 4get/KN2 reporter (*Il4*^*4get/KN2*^) mice, percentage hCD2^+^ cells was calculated by percentage hCD2^+^ minus percentage hCD2^+^ in non-KN2 reporter mice (*Il4*^*4get/4get*^) or, in some experiments by percentage hCD2^+^ minus percentage isotype control positive.

Gating strategies are shown in the Figures or in Supporting Materials (lymphocyte populations in the BAL fluid and lungs of GREAT and SMART-17A reporter mice (S6 Fig), 4get reporter^+^ cells in the lungs (S7 Fig), non-lymphocyte populations in the BAL fluid and lungs (S8 Fig), and flow cytometry-sorted cells (S9 Fig)).

### Enrichment of LECs and lung CD45^+^ cells

LECs and CD45^+^ hematopoietic cells were enriched using a modified protocol described in Sinha and Lowell [67]. Briefly, lungs were perfused with 5 ml D-PBS via the cardiac right ventricle. After cannulating the trachea, 1.2 ml of Dispase II (50 U/ml, Roche) solution, followed by 0.6 ml of 1% low-melt agarose (Fisher Scientific, Waltham, MA) were instilled into the lungs. Ice was placed on the lungs for 2 min to solidify the agarose. Whole lungs, but not the trachea, were then dissected out, rinsed in D-PBS, then placed in a 50 ml conical tube with 0.5 ml Dispase II, and digested for 45 min at RT, with shaking at 150 rpm. Lung cells were released by gentle and repeated teasing of the parenchymal tissue with forceps. After sequentially straining of the cell suspension through 70, 45 and 20 μm strainers, the cells were centrifuged, resuspended in medium, and stained with the following biotinylated antibodies for 45 min on ice: CD45 (30F11), Ter119, CD31 (MEC13.3), and CD16/32 (2.4G2). After washing, cells were incubated with Dynabeads MyOne Streptavidin T1 (Life Technologies) (3 μl/10^6^ cells), that were pre-rinsed with D-PBS, on a rotator for 30 min at 4°C. Afterwards, they were separated into CD45^+^ and LEC fractions with a DynaMag-2 magnet (Life Technologies). In the LEC fraction, ~78% of the cells, on average, were positive for the epithelial cell adhesion molecule (EpCAM), a pan-epithelial cell marker (S10A Fig). This fraction was also minimally contaminated with CD45 positive cell (~0.53%). Of the EpCAM^+^ cells, ~88% were alveolar type II cells (intracellular staining of surfactant protein C). The remaining EpCAM^+^ cells were most likely club cells (previously known as Clara cells) and distal lung progenitor cells. Compared to whole lungs, the CD45 fraction was enriched with mRNA encoding CD45 (*Ptprc*) and depleted of mRNA encoding EpCAM (S10B Fig).

### RNA isolation and quantitative PCR

Total RNA was isolated from whole lungs, enriched LECs, and CD45^+^ cells using RNeasy Mini Kit (Qiagen, Valencia, CA), with on-column DNase digestion. Total RNA was isolated from sorted cells using RNeasy Micro Plus Kit (Qiagen), using a genomic DNA removal column. RNA was reverse transcribed to generate cDNA using iScript cDNA synthesis kit (Bio-Rad). In most experiments, RNA was quantified using iTaq Universal SYBR Green Supermix (Bio-Rad) on a Step-One Plus real-time PCR machine (Life Technologies) using the primers in Table 1. In some experiments, RNA was quantified using Taqman Gene Expression Assays and Taqman Universal Master Mix II with UNG (both from Life Technologies). The experiments that used Taqman Expression Assay were as follows: *Ccl20* (Fig 6E), *Il12a* (Fig 4C and 4I), *Il23a* (Fig 6C, 6E and 6F), and *Tslp* (Fig 6E). Quantification cycles (Cq) of the gene of interest (GOI) were normalized to *Hprt* (ΔCq = Cq_GOI_-Cq_Hprt_), and relative expression (2^-ΔCq^) was calculated and plotted.

**Table 1:** Primer pairs for quantitative PCR.

	Forward primer	Reverse Primer
*Ccl20*	GCCTCTCGTACATACAGACGC	CCAGTTCTGCTTTGGATCAGC
*Csf2*	AGCAGGGTCTACGGGGC	TGAAATCCGCATAGGTGG
*Epcam*	GGTGGTGTCATTAGCAGTCA	GGATCTCACCCATCTCCTTTATC
*Hprt*	AGGTTGCAAGCTTGCTGGT	TGAAGTACTCATTATAGTCAAGGGCA
*Il1a*	CCCATGATCTGGAAGAGACCA	CAAACTTCTGCCTGACGAGC
*Il1b*	GCCACCTTTTGACAGTGATGAG	GACAGCCCAGGTCAAAGGTT
*Il12b*	TGCTGGTGTCTCCACTCAT	CTTCAGGCGTGTCACAGG
*Il23a*	TATCCAGTGTGAAGATGGTTGTG	CACTAAGGGCTCAGTCAGAGTTG
*Il33*	GCTGCGTCTGTTGACACATTGAG	GGTCTTGCTCTTGGTCTTTTCCAG
*Il6*	GTTCTCTGGGAAATCGTGGA	TGTACTCCAGGTAGCTATGG
*Tnfa*	TCTTCTGTCTACTGAACTTCGGGGT	GGCCATAGAACTGATGAGAGGG
*Ptprc*	TTCCAAGAGGAAGGAGCCCA	AGAACAACCCTGTCTGCTGG
*Tslp*	TCGAGGACTGTGAGAGCAAGCCAG	CTGGAGATTGCATGAAGGAATACCA

### IL-33 protein quantification

Protein extracts of whole lungs were made after snap freezing treated lungs in liquid nitrogen, followed by homogenization using a disperser/homogenizer (IKA, Wilmington, NC) in 500 μl TNT (0.1 M Tris-Cl, 150 mM NaCl, 0.1% Tween 20) lysis buffer and cOmplete mini protease inhibitors (Roche). The amounts of mature IL-33 protein were assayed using Mouse IL-33 DuoSet ELISA Kit (R&D Systems, Minneapolis, MN) according to the manufacturer’s instructions.

### Statistical tests

Statistics were performed using Prism 5 (GraphPad Software, La Jolla, CA) as described in the Figure Legends. Outliers identified using Grubb’s test after pooling data from all experiments of one type were removed from analysis. The data from the following mice were removed from analysis: 1 OVA-treated mouse in Fig 2D–2F, and 1 OVA-treated mouse in Fig 4K.

## Supporting Information

S1 Fig Flagellin leads to increased eosinophils in the lung. Mice were administered with OVA (O), OVA plus flagellin (1 μg), or OVA plus CpG (3 μg) i.n., and challenged with i.n. OVA. On d21, numbers of eosinophils were assessed in the lung. Data contain four mice per group and are representative of one of three independent experiments. Error bars indicate mean +SD. * P ≤ 0.05 using one-way anova with Bonferroni post-test.
(TIF)

S2 Fig I.n. sensitization with OVA plus CpG ODN does not lead to increased pulmonary resistance. Mice were sensitized with OVA plus 3 μg CpG i.n. and challenged with i.n. OVA. On d22, pulmonary resistance was assessed. Data are pooled from two independent experiments with 12 mice total per group. Error bars indicate mean +SD.
(TIF)

S3 Fig Flagellin and CpG ODN induce IL-17 production in γδ and CD4 T cells during the innate immune response. Percentages of CD4 T cells, γδ T cells, and iNKT cells producing IL-17A (hNGFR^+^) in SMART-17A reporter mice (SMART-17A) one day after third i.n. administration (d3) of OVA, OVA plus flagellin (1 μg), or OVA plus CpG (3 μg). Data are pooled from three independent experiments with combined totals of 10 or 12 mice per group. Error bars indicate mean +SD. * P ≤ 0.05, ** P ≤ 0.01, *** P ≤ 0.001 using one-way anova with Bonferroni post-test.
(TIF)

S4 Fig Both CD103^+^ and CD11b^+^ migratory DCs upregulate activation markers to i.n. exposure to flagellin or CpG ODN. Expression of activation markers on migratory DCs in the lung-draining (mediastinal) LNs of *Myd88*^*fl/fl*^ (*M*^*F*^) one day after i.n. administration (d1) of OVA-AF647 or OVA-AF647 plus TLR ligand. (**A**) Migratory DCs were gated as CD11c^+^I-A^b(hi)^, then gated on CD103 and CD11b. (**B**) Comparison of different activation markers on migratory DC subsets that have taken up OVA in *M*^*F*^ mice treated i.n with OVA-AF647, OVA-AF647 plus flagellin (1 μg), or OVA-AF647 plus CpG (0.75 or 3 μg). Data contain 3–4 mice per group and are representative of at least 3 independent experiments. Error bars indicate mean +SD. * P ≤ 0.05, ** P ≤ 0.01, *** P ≤ 0.001 using one-way anova with Bonferroni post-test.
(TIF)

S5 Fig Defining live cells for flow cytometry analysis and cell sorting. (**A**) For flow cytometry analysis and cell sorting of lung and BAL fluid cell suspensions, DAPI^-^ and DAPI^int^ cells were gated as “live”. (**B**) In subsequent gating, other cell types were then identified as “live” based on lack of staining with DAPI.
(TIF)

S6 Fig Gating strategies for defining lymphocyte populations from the BAL fluid and the lungs of GREAT and SMART-17A reporter mice. (A) Lymphocytes in the BAL fluid (Fig 1B) were identified as SiglecF^-^, then gated as followed: B cells (B220^+^TCRβ^-^), NK cells (CD49b^+^B220^-^TCRβ^-^ and GFP^-^ to exclude basophils in *Il4*^*4get/4get*^ mice [31]), CD4 T cells (TCRβ^+^CD4^+^B220^-^CD8^-^), and CD8 T cells (TCRβ^+^CD8^+^B220^-^CD4^-^) (B) Gating strategy for defining lymphocyte populations using CD1d-tetramer (CD1d-tet) to identify invariant (i) NKT cells in the experiments shown in Fig 2G–2I, Fig 4D and 4E, and Fig 4J and 4K. Cells were identified by the following cell surface markers: iNKT cells (CD1d-tet^+^CD3^+^), NK cells (NK1.1^+^CD3^-^CD1d-tet^-^TCRγδ^-^), γδ T cells (TCRγδ^+^CD3^+^CD1d-tet^-^), CD4 T cells (CD4^+^CD3^+^CD1d-tet^-^TCRγδ^-^CD8^-^), and CD8 T cells (CD8^+^CD3^+^CD1d-tet^-^TCRγδ^-^CD4^-^). (C) Gating strategy for defining lymphocytes using NK1.1 and CD3 to identify NKT cells in the experiments shown in Fig 2D–2F and Fig 4A. For these experiments, cells were identified by the following cell surface markers: γδ T cells (TCRγδ^+^CD3^+^), NK cells (NK1.1^+^TCRγδ^-^CD3^-^), NKT cells (NK1.1^+^CD3^+^TCRγδ^-^), CD4 T cells (CD4^+^CD3^+^TCRγδ^-^NK1.1^-^CD8^-^), and CD8 T cells (CD8^+^CD3^+^CD1d-tet^-^TCRγδ^-^NK1.1^-^CD4^-^).
(TIF)

S7 Fig Gating strategy for 4get reporter^+^ cells in the lung. Gating strategy for 4get reporter^+^ CD4 T cells and basophils in the lungs of 4get/KN2 reporter mice as shown in Fig 2A–2C. Cells were identified by using the following markers: 4get^+^(GFP^+^) CD4 T cells (GFP^+^CD4^+^CD3^+^CD1d-tet^-^) and basophils (GFP^+^CD49b^+^SSC^lo^CD3^-^CD1d-tet^-^CD4^-^). Basophils and eosinophils are constitutively 4get^+^ [31]. The gating strategy shown is from *Myd88*^*fl/+*^ *Il4*^*4get/KN2*^ *Mcpt8*^*Basopho8/+*^ mice. *Mcpt8*^*Basopho8*^ mice express both YFP and Cre in basophils [61]. Both GFP from 4get reporter and YFP from Basopho8 reporter were read using the same filter/channel on the flow cytometer, and additional markers were used to distinguish basophils as described above.
(TIF)

S8 Fig Gating strategy for non-lymphocyte populations in the lung and BAL fluid. Gating strategy for identifying non-lymphocyte populations in the lung and BAL fluid in experiments shown in Fig 1B and Fig 3A–3C. Cells were identified by using the following cell surface markers: eosinophils (SiglecF^+^CD11b^+^CD11c^-^Ly6G^-^), neutrophils (Ly6G^+^Ly6C^+^CD11b^+^), and monocytes (Ly6C^+^CD11b^+^CD11c^-/int^SiglecF^-^Ly6G^-^).
(TIF)

S9 Fig Gating strategy for cell-sorted cells. Gating strategy for experiments shown in For Figs 4H and 4I and 6F. (**A**) AMs, CD103^+^ cDCs, CD11b^+^ cDCs, and moDCs from CD11c-enriched cell suspensions were sorted as follows: AMs (SiglecF^+^CD11c^+^B220^-^CD3^-^NK1.1^-^Ly6G^-^), CD103^+^ cDCs (CD11c^+^MHCII^+^CD103^+^B220^-^CD3^-^NK1.1^-^Ly6G^-^SiglecF^-^) CD11b^+^ cDCs (CD11c^+^MHCII^+^CD11b^+^CD64^-^B220^-^CD3^-^NK1.1^-^Ly6G^-^SiglecF^-^), andmoDCs (CD11c^+^MHCII^+^CD11b^+^CD64^+^B220^-^CD3^-^NK1.1^-^Ly6G^-^SiglecF^-^). (**B**) Ly6C^hi^ monocytes from CD11c-depleted lung cell suspension were sorted as follows: Ly6C^hi^ CD11b^+^SiglecF^-^CD11c^-^CD19^-^CD3^-^ CD64^-^ Ly6G^-^NK1.1^-^
(TIF)

S10 Fig Enrichment of LECs and CD45 cell fractions. Cell enrichments were assessed after the LEC and CD45 cell separation from the lung. (**A**) Representative flow cytometry plots of LEC fraction stained with CD45, EpCAM, and SPC, and percentages EpCAM^+^ of LEC fraction, percentages SPC^+^ of EpCAM^+^ LEC fraction, and percentages of CD45^+^ of LEC fraction. (**B**) *Epcam* and *Ptprc* RNA analysis of CD45 fraction as compared to whole lung. Data contain 3–4 mice per group and are representative of two independent experiments. Error bars indicate mean +SD.
(TIF)

## References

[1] Schroder K, Hertzog PJ, Ravasi T, Hume DA. Interferon-gamma: an overview of signals, mechanisms and functions. J Leukoc Biol. 2004;75: 163–89 10.1189/jlb.0603252 14525967

[2] Pulendran B, Artis D. New paradigms in type 2 immunity. Science. 2012;337: 431–5. 10.1126/science.1221064 22837519 PMC4078898

[3] Iwasaki A, Medzhitov R. Toll-like receptor control of the adaptive immune responses. Nat Immunol. 2004;5: 987–95. 10.1038/ni1112 15454922

[4] Hou B, Reizis B, DeFranco AL. Toll-like receptors activate innate and adaptive immunity by using dendritic cell-intrinsic and -extrinsic mechanisms. Immunity. 2008;29: 272–82 10.1016/j.immuni.2008.05.016 18656388 PMC2847796

[5] Spörri R, Reis e Sousa C. Inflammatory mediators are insufficient for full dendritic cell activation and promote expansion of CD4+ T cell populations lacking helper function. Nat Immunol. 2005;6: 163–70 10.1038/ni1162 15654341

[6] Trinchieri G. Interleukin-12 and the regulation of innate resistance and adaptive immunity. Nat Rev Immunol. 2003;3: 133–46 10.1038/nri1001 12563297

[7] Szabo SJ, Sullivan BM, Peng SL, Glimcher LH. Molecular mechanisms regulating Th1 immune responses. Annu Rev Immunol. 2003;21: 713–58. 10.1146/annurev.immunol.21.120601.140942 12500979

[8] Lambrecht BN, Hammad H. Allergens and the airway epithelium response: Gateway to allergic sensitization. J Allergy Clin Immunol. Elsevier Ltd; 2014;134: 499–507 10.1016/j.jaci.2014.06.036 25171864

[9] Saenz SA, Taylor BC, Artis D. Welcome to the neighborhood: Epithelial cell-derived cytokines license innate and adaptive immune responses at mucosal sites. Immunol Rev. 2008;226: 172–190 10.1111/j.1600-065X.2008.00713.x 19161424 PMC2683382

[10] Besnard A-G, Togbe D, Guillou N, Erard F, Quesniaux V, Ryffel B. IL-33-activated dendritic cells are critical for allergic airway inflammation. Eur J Immunol. 2011;41: 1675–86. 10.1002/eji.201041033 21469105

[11] Rank MA, Kobayashi T, Kozaki H, Bartemes KR, Squillace DL, Kita H. IL-33-activated dendritic cells induce an atypical TH2-type response. J Allergy Clin Immunol. Elsevier Ltd; 2009;123: 1047–54. 10.1016/j.jaci.2009.02.026 19361843 PMC2711963

[12] Soumelis V, Reche PA, Kanzler H, Yuan W, Edward G, Homey B, et al Human epithelial cells trigger dendritic cell mediated allergic inflammation by producing TSLP. Nat Immunol. 2002;3: 673–80. 10.1038/ni805 12055625

[13] Willart MAM, Deswarte K, Pouliot P, Braun H, Beyaert R, Lambrecht BN, et al Interleukin-1α controls allergic sensitization to inhaled house dust mite via the epithelial release of GM-CSF and IL-33. J Exp Med. 2012;209: 1505–17. 10.1084/jem.20112691 22802353 PMC3409497

[14] Hammad H, Lambrecht BN. Dendritic cells and epithelial cells: linking innate and adaptive immunity in asthma. Nat Rev Immunol. 2008;8: 193–204. 10.1038/nri2275 18301423

[15] Dinarello CA. Immunological and inflammatory functions of the interleukin-1 family. Annu Rev Immunol. 2009;27: 519–50. 10.1146/annurev.immunol.021908.132612 19302047

[16] Sanders CJ, Moore DA, Williams IR, Gewirtz AT. Both radioresistant and hemopoietic cells promote innate and adaptive immune responses to flagellin. J Immunol. 2008;180: 7184–92. Available: http://www.jimmunol.org/content/180/11/7184.short 18490717 10.4049/jimmunol.180.11.7184

[17] Hammad H, Chieppa M, Perros F, Willart MA, Germain RN, Lambrecht BN. House dust mite allergen induces asthma via Toll-like receptor 4 triggering of airway structural cells. Nat Med. 2009;15: 410–6. 10.1038/nm.1946 19330007 PMC2789255

[18] Trompette A, Divanovic S, Visintin A, Blanchard C, Hegde RS, Madan R, et al Allergenicity resulting from functional mimicry of a Toll-like receptor complex protein. Nature. 2009;457: 585–8. 10.1038/nature07548 19060881 PMC2843411

[19] Hayashi F, Smith KD, Ozinsky A, Hawn TR, Yi EC, Goodlett DR, et al The innate immune response to bacterial flagellin is mediated by Toll-like receptor 5. Nature. 2001;410: 1099–103. 10.1038/35074106 11323673

[20] Wilson RH, Maruoka S, Whitehead GS, Foley JF, Flake GP, Sever ML, et al The Toll-like receptor 5 ligand flagellin promotes asthma by priming allergic responses to indoor allergens. Nat Med. Nature Publishing Group; 2012;18: 1705–10. 10.1038/nm.2920 23064463 PMC3493750

[21] Herrick CA, MacLeod H, Glusac E, Tigelaar RE, Bottomly K. Th2 responses induced by epicutaneous or inhalational protein exposure are differentially dependent on IL-4. J Clin Invest. 2000;105: 765–75. 10.1172/JCI8624 10727445 PMC377464

[22] Eisenbarth SC, Piggott DA, Huleatt JW, Visintin I, Herrick CA, Bottomly K. Lipopolysaccharide-enhanced, toll-like receptor 4-dependent T helper cell type 2 responses to inhaled antigen. J Exp Med. 2002;196: 1645–51. 10.1084/jem.20021340 12486107 PMC2196061

[23] Van Maele L, Fougeron D, Janot L, Didierlaurent A, Cayet D, Tabareau J, et al Airway structural cells regulate TLR5-mediated mucosal adjuvant activity. Mucosal Immunol. Nature Publishing Group; 2014;7: 489–500. 10.1038/mi.2013.66 24064672

[24] Chen L, Arora M, Yarlagadda M, Oriss TB, Krishnamoorthy N, Ray A, et al Distinct responses of lung and spleen dendritic cells to the TLR9 agonist CpG oligodeoxynucleotide. J Immunol. 2006;177: 2373–83. Available: http://www.ncbi.nlm.nih.gov/pubmed/16887999 16887999 10.4049/jimmunol.177.4.2373

[25] Pesce I, Monaci E, Muzzi A, Tritto E, Tavarini S, Nuti S, et al Intranasal administration of CpG induces a rapid and transient cytokine response followed by dendritic and natural killer cell activation and recruitment in the mouse lung. J Innate Immun. 2010;2: 144–59. 10.1159/000254948 20375632

[26] Didierlaurent A, Ferrero I, Otten L a., Dubois B, Reinhardt M, Carlsen H, et al Flagellin promotes myeloid differentiation factor 88-dependent development of Th2-type response. J Immunol. 2004;172: 6922–30. 15153511 10.4049/jimmunol.172.11.6922

[27] Cunningham AF, Khan M, Ball J, Toellner K-M, Serre K, Mohr E, et al Responses to the soluble flagellar protein FliC are Th2, while those to FliC on Salmonella are Th1. Eur J Immunol. 2004;34: 2986–95. 10.1002/eji.200425403 15384042

[28] Krieg AM. CpG motifs in bacterial DNA and their immune effects. Annu Rev Immunol. 2002;20: 709–60. 10.1146/annurev.immunol.20.100301.064842 11861616

[29] Paul WE, Zhu J. How are T(H)2-type immune responses initiated and amplified? Nat Rev Immunol. Nature Publishing Group; 2010;10: 225–35. 10.1038/nri2735 20336151 PMC3496776

[30] Mohrs M, Shinkai K, Mohrs K, Locksley RM. Analysis of type 2 immunity in vivo with a bicistronic IL-4 reporter. Immunity. 2001;15: 303–11. Available: http://www.sciencedirect.com/science/article/pii/S1074761301001868 11520464 10.1016/s1074-7613(01)00186-8

[31] Voehringer D, Shinkai K, Locksley RM. Type 2 immunity reflects orchestrated recruitment of cells committed to IL-4 production. Immunity. 2004;20: 267–77. Available: http://www.ncbi.nlm.nih.gov/pubmed/15030771 15030771 10.1016/s1074-7613(04)00026-3

[32] Mohrs K, Wakil AE, Killeen N, Locksley RM, Mohrs M. A two-step process for cytokine production revealed by IL-4 dual-reporter mice. Immunity. 2005;23: 419–29. 10.1016/j.immuni.2005.09.006 16226507 PMC2826320

[33] Reinhardt RL, Liang H-E, Locksley RM. Cytokine-secreting follicular T cells shape the antibody repertoire. Nat Immunol. 2009;10: 385–93. 10.1038/ni.1715 19252490 PMC2714053

[34] Price AE, Reinhardt RL, Liang H-E, Locksley RM. Marking and quantifying IL-17A-producing cells in vivo. PLoS One. 2012;7: e39750 10.1371/journal.pone.0039750 22768117 PMC3387253

[35] Feuillet V, Medjane S, Mondor I, Demaria O, Pagni PP, Galán JE, et al Involvement of Toll-like receptor 5 in the recognition of flagellated bacteria. Proc Natl Acad Sci U S A. 2006;103: 12487–12492. 10.1073/pnas.0605200103 16891416 PMC1567905

[36] Fougeron D, Van Maele L, Songhet P, Cayet D, Hot D, Van Rooijen N, et al Indirect Toll-like receptor 5-mediated activation of conventional dendritic cells promotes the mucosal adjuvant activity of flagellin in the respiratory tract. Vaccine. Elsevier Ltd; 2015; 1–11. 10.1016/j.vaccine.2015.05.022 26003491

[37] Honko AN, Mizel SB. Mucosal administration of flagellin induces innate immunity in the mouse lung. Infect Immun. 2004;72: 6676–9. 10.1128/IAI.72.11.6676-6679.2004 15501801 PMC523048

[38] Lamkanfi M, Dixit VM. Mechanisms and functions of inflammasomes. Cell. Elsevier Inc.; 2014;157: 1013–22. 10.1016/j.cell.2014.04.007 24855941

[39] Mathur R, Oh H, Zhang D, Park S-G, Seo J, Koblansky A, et al A mouse model of Salmonella typhi infection. Cell. Elsevier Inc.; 2012;151: 590–602. 10.1016/j.cell.2012.08.042 23101627 PMC3500584

[40] Kawai T, Akira S. Toll-like receptors and their crosstalk with other innate receptors in infection and immunity. Immunity. Elsevier Inc.; 2011;34: 637–50. 10.1016/j.immuni.2011.05.006 21616434

[41] O’Neill LAJ. The interleukin-1 receptor/Toll-like receptor superfamily: 10 Years of progress. Immunological Reviews. 2008 pp. 10–18. 10.1111/j.1600-065X.2008.00701.x 19161412

[42] Abram CL, Roberge GL, Hu Y, Lowell CA. Comparative analysis of the efficiency and specificity of myeloid-Cre deleting strains using ROSA-EYFP reporter mice. J Immunol Methods. Elsevier B.V.; 2014;408: 89–100. 10.1016/j.jim.2014.05.009 24857755 PMC4105345

[43] Crotty S. Follicular helper CD4 T cells (TFH). Annu Rev Immunol. 2011;29: 621–63. 10.1146/annurev-immunol-031210-101400 21314428

[44] MacDonald AS, Maizels RM. Alarming dendritic cells for Th2 induction. J Exp Med. 2008;205: 13–7. 10.1084/jem.20072665 18195077 PMC2234366

[45] Liu Y-J. Thymic stromal lymphopoietin and OX40 ligand pathway in the initiation of dendritic cell-mediated allergic inflammation. J Allergy Clin Immunol. 2007;120: 238–44–6. 10.1016/j.jaci.2007.06.004 17666213

[46] Sokol CL, Barton GM, Farr AG, Medzhitov R. A mechanism for the initiation of allergen-induced T helper type 2 responses. Nat Immunol. 2008;9: 310–8. 10.1038/ni1558 18300366 PMC3888112

[47] Bartemes KR, Kita H. Dynamic role of epithelium-derived cytokines in asthma. Clin Immunol. Elsevier Inc.; 2012;143: 222–235. 10.1016/j.clim.2012.03.001 22534317 PMC3358585

[48] Janot L, Sirard J-C, Secher T, Noulin N, Fick L, Akira S, et al Radioresistant cells expressing TLR5 control the respiratory epithelium’s innate immune responses to flagellin. Eur J Immunol. 2009;39: 1587–96. 10.1002/eji.200838907 19424969

[49] Hawn TR, Berrington WR, Smith IA, Uematsu S, Akira S, Aderem A, et al Altered inflammatory responses in TLR5-deficient mice infected with Legionella pneumophila. J Immunol. 2007;179: 6981–7. Available: http://www.jimmunol.org/content/179/10/6981.short 17982089 10.4049/jimmunol.179.10.6981

[50] Suzuki K, Suda T, Naito T, Ide K, Chida K, Nakamura H. Impaired toll-like receptor 9 expression in alveolar macrophages with no sensitivity to CpG DNA. Am J Respir Crit Care Med. 2005;171: 707–13. 10.1164/rccm.200408-1078OC 15640365

[51] Nakano H, Free ME, Whitehead GS, Maruoka S, Wilson RH, Nakano K, et al Pulmonary CD103(+) dendritic cells prime Th2 responses to inhaled allergens. Mucosal Immunol. Nature Publishing Group; 2012;5: 53–65. 10.1038/mi.2011.47 22012243 PMC3697034

[52] Tussiwand R, Everts B, Grajales-Reyes GE, Kretzer NM, Iwata A, Bagaitkar J, et al Klf4 Expression in Conventional Dendritic Cells Is Required for T Helper 2 Cell Responses. Immunity. Elsevier Inc.; 2015;42: 916–928. 10.1016/j.immuni.2015.04.017 25992862 PMC4447135

[53] Plantinga M, Guilliams M, Vanheerswynghels M, Deswarte K, Branco-Madeira F, Toussaint W, et al Conventional and Monocyte-Derived CD11b+ Dendritic Cells Initiate and Maintain T Helper 2 Cell-Mediated Immunity to House Dust Mite Allergen. Immunity. Elsevier Inc.; 2013;38: 322–335. 10.1016/j.immuni.2012.10.016 23352232

[54] Shim J-U, Lee SE, Hwang W, Lee C, Park J-W, Sohn J-H, et al Flagellin suppresses experimental asthma by generating regulatory dendritic cells and T cells. J Allergy Clin Immunol. 2016;137: 426–35. 10.1016/j.jaci.2015.07.010 26303344

[55] Schülke S, Waibler Z, Mende M-S, Zoccatelli G, Vieths S, Toda M, et al Fusion protein of TLR5-ligand and allergen potentiates activation and IL-10 secretion in murine myeloid DC. Mol Immunol. 48: 341–50. 10.1016/j.molimm.2010.07.006 20965571

[56] Schülke S, Burggraf M, Waibler Z, Wangorsch A, Wolfheimer S, Kalinke U, et al A fusion protein of flagellin and ovalbumin suppresses the TH2 response and prevents murine intestinal allergy. J Allergy Clin Immunol. 2011;128: 1340–1348.e12. 10.1016/j.jaci.2011.07.036 21872305

[57] McAlees JW, Whitehead GS, Harley IT, Cappelletti M, Rewerts CL, Holdcroft a M, et al Distinct Tlr4-expressing cell compartments control neutrophilic and eosinophilic airway inflammation. Mucosal Immunol. Nature Publishing Group; 2014; 10.1038/mi.2014.117 PMC4454628 25465099

[58] Lambrecht BN, Hammad H. The immunology of asthma. Nat Immunol. 2014;16: 45–56. 10.1038/ni.3049 25521684

[59] Thomas WR, Hales BJ, Smith W-A. Structural biology of allergens. Curr Allergy Asthma Rep. 2005;5: 388–393. 16091212 10.1007/s11882-005-0012-1

[60] Clausen BE, Burkhardt C, Reith W, Renkawitz R, Förster I. Conditional gene targeting in macrophages and granulocytes using LysMcre mice. Transgenic Res. 1999;8: 265–77. Available: http://www.ncbi.nlm.nih.gov/pubmed/10621974 10621974 10.1023/a:1008942828960

[61] Sullivan BM, Liang H-E, Bando JK, Wu D, Cheng LE, McKerrow JK, et al Genetic analysis of basophil function in vivo. Nat Immunol. 2011;12: 527–35. 10.1038/ni.2036 21552267 PMC3271435

[62] Zhang D, Zhang G, Hayden MS, Greenblatt MB, Bussey C, Flavell R a, et al A toll-like receptor that prevents infection by uropathogenic bacteria. Science. 2004;303: 1522–6. 10.1126/science.1094351 15001781

[63] Aida Y, Pabst MJ. Removal of endotoxin from protein solutions by phase separation using Triton X-114. J Immunol Methods. 1990;132: 191–5. 2170533 10.1016/0022-1759(90)90029-u

[64] Smith KD, Andersen-Nissen E, Hayashi F, Strobe K, Bergman MA, Barrett SLR, et al Toll-like receptor 5 recognizes a conserved site on flagellin required for protofilament formation and bacterial motility. Nat Immunol. 2003;4: 1247–1253. 10.1038/ni1011 14625549

[65] Bhattacharya M, Sundaram A, Kudo M, Farmer J, Ganesan P, Khalifeh-Soltani A, et al IQGAP1-dependent scaffold suppresses RhoA and inhibits airway smooth muscle contraction. J Clin Invest. 2014;124: 4895–8. 10.1172/JCI76658 25271629 PMC4347230

[66] Nakano H, Cook DN. Pulmonary antigen presenting cells: isolation, purification, and culture. Methods Mol Biol. 2013;1032: 19–29. 10.1007/978-1-62703-496-8_2 23943441 PMC5552037

[67] Sinha M, Lowell CA. Immune defense protein expression in highly purified mouse lung epithelial cells. Am J Respir Cell Mol Biol. 2015; 1–48. 10.1165/rcmb.2015-0171OC PMC4942217 26574781

