# Supplementary figures and images for: Determinants of Divergent Adaptive Immune Responses after Airway Sensitization with Ligands of Toll-Like Receptor 5 or Toll-Like Receptor 9
Source: PLoS One. 2016 Dec 15;11(12):e0167693. doi: 10.1371/journal.pone.0167693 (PMC5157987; doi:10.1371/journal.pone.0167693)

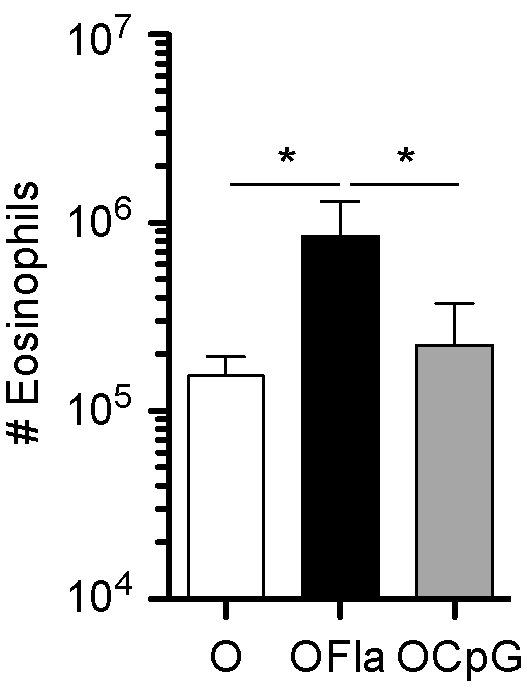

Supplement: S1 Fig — Mice were administered with OVA (O), OVA plus flagellin (1 μg), or OVA plus CpG (3 μg) i.n., and challenged with i.n. OVA. On d21, numbers of eosinophils were assessed in the lung. Data contain four mice per group and are representative of one of three independent experiments. Error bars indicate mean +SD. * P ≤ 0.05 using one-way anova with Bonferroni post-test. (TIF) [file pone.0167693.s001.tif]

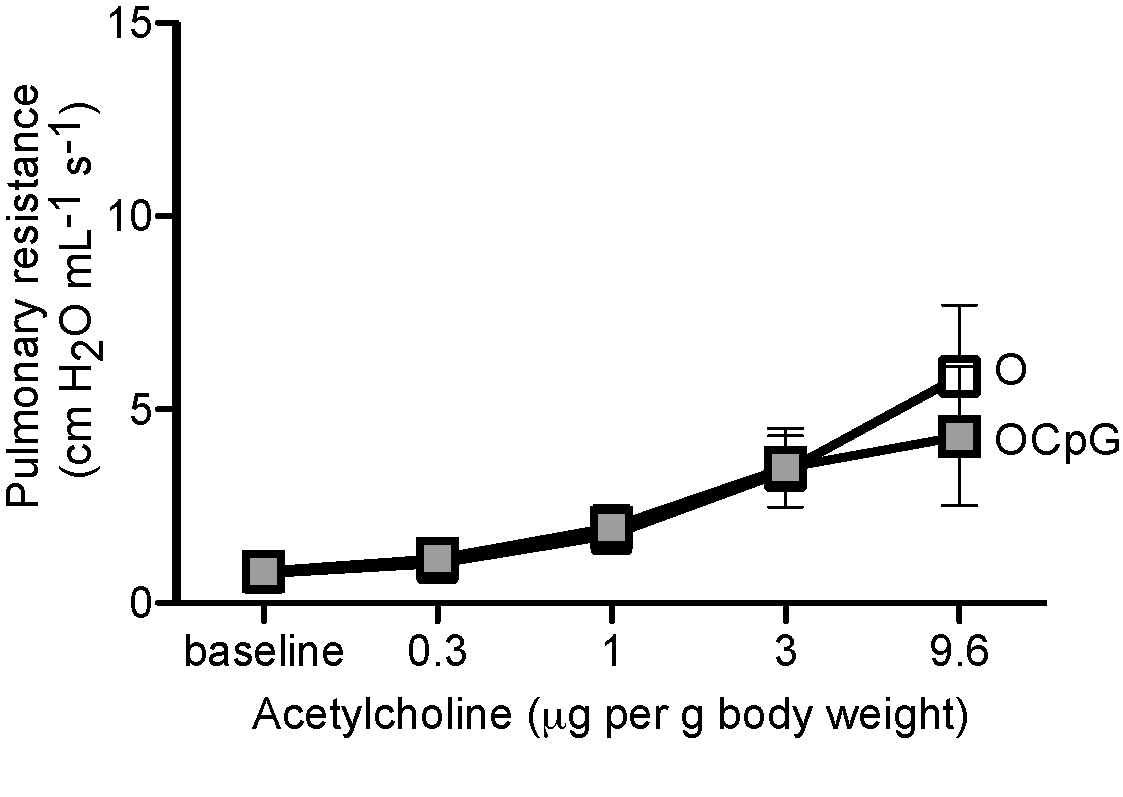

Supplement: S2 Fig — Mice were sensitized with OVA plus 3 μg CpG i.n. and challenged with i.n. OVA. On d22, pulmonary resistance was assessed. Data are pooled from two independent experiments with 12 mice total per group. Error bars indicate mean +SD. (TIF) [file pone.0167693.s002.tif]

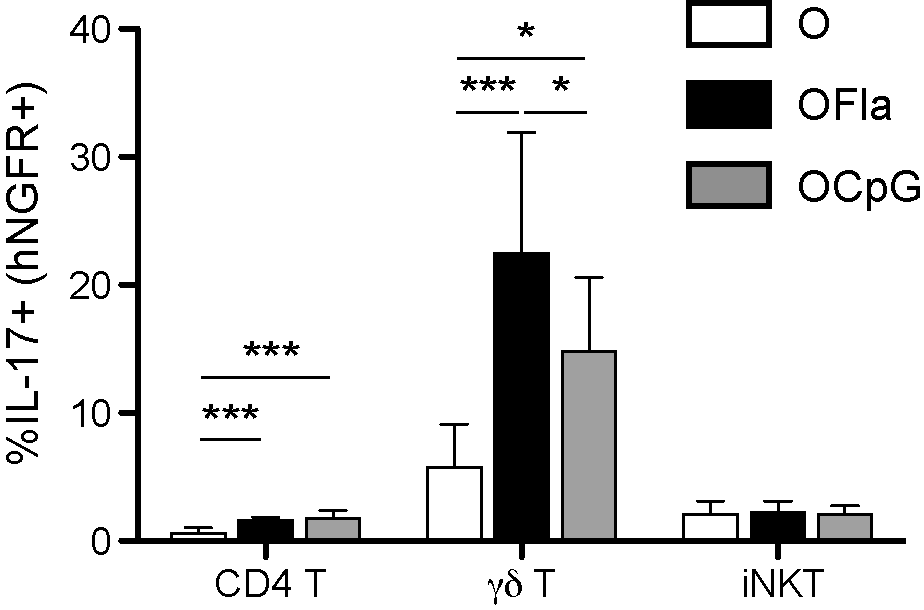

Supplement: S3 Fig — Percentages of CD4 T cells, γδ T cells, and iNKT cells producing IL-17A (hNGFR+) in SMART-17A reporter mice (SMART-17A) one day after third i.n. administration (d3) of OVA, OVA plus flagellin (1 μg), or OVA plus CpG (3 μg). Data are pooled from three independent experiments with combined totals of 10 or 12 mice per group. Error bars indicate mean +SD. * P ≤ 0.05, ** P ≤ 0.01, *** P ≤ 0.001 using one-way anova with Bonferroni post-test. (TIF) [file pone.0167693.s003.tif]

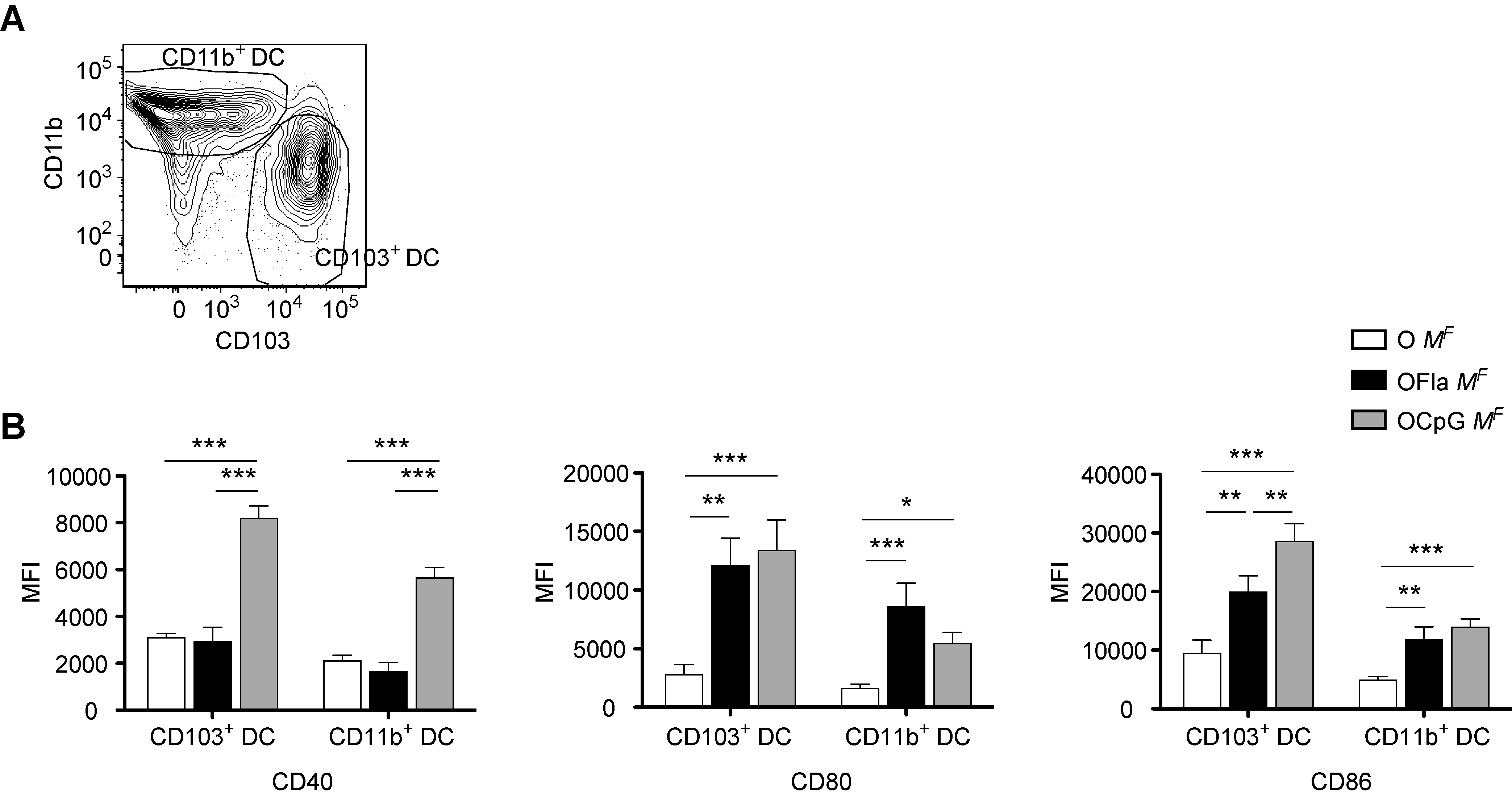

Supplement: S4 Fig — Expression of activation markers on migratory DCs in the lung-draining (mediastinal) LNs of Myd88fl/fl (MF) one day after i.n. administration (d1) of OVA-AF647 or OVA-AF647 plus TLR ligand. (A) Migratory DCs were gated as CD11c+I-Ab(hi), then gated on CD103 and CD11b. (B) Comparison of different activation markers on migratory DC subsets that have taken up OVA in MF mice treated i.n with OVA-AF647, OVA-AF647 plus flagellin (1 μg), or OVA-AF647 plus CpG (0.75 or 3 μg). Data contain 3–4 mice per group and are representative of at least 3 independent experiments. Error bars indicate mean +SD. * P ≤ 0.05, ** P ≤ 0.01, *** P ≤ 0.001 using one-way anova with Bonferroni post-test. (TIF) [file pone.0167693.s004.tif]

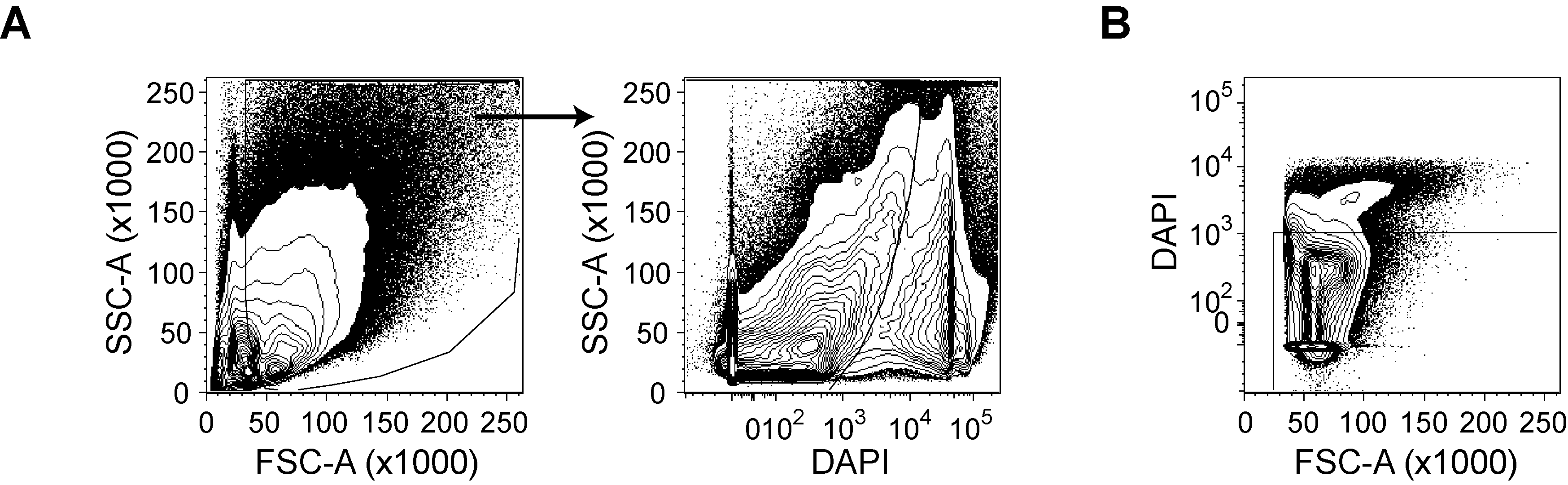

Supplement: S5 Fig — (A) For flow cytometry analysis and cell sorting of lung and BAL fluid cell suspensions, DAPI- and DAPIint cells were gated as “live”. (B) In subsequent gating, other cell types were then identified as “live” based on lack of staining with DAPI. (TIF) [file pone.0167693.s005.tif]

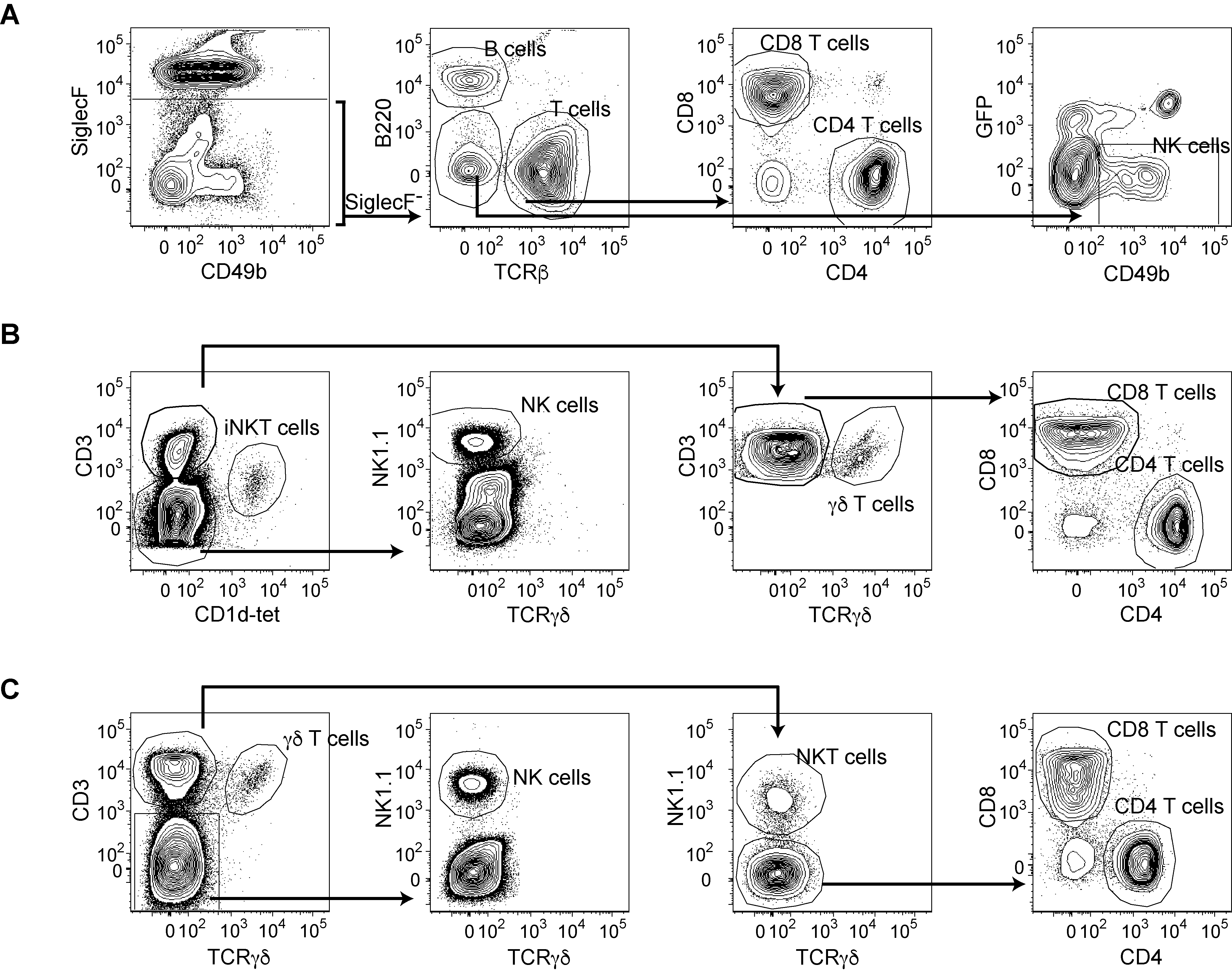

Supplement: S6 Fig — (A) Lymphocytes in the BAL fluid (Fig 1B) were identified as SiglecF-, then gated as followed: B cells (B220+TCRβ-), NK cells (CD49b+B220-TCRβ- and GFP- to exclude basophils in Il44get/4get mice [31]), CD4 T cells (TCRβ+CD4+B220-CD8-), and CD8 T cells (TCRβ+CD8+B220-CD4-) (B) Gating strategy for defining lymphocyte populations using CD1d-tetramer (CD1d-tet) to identify invariant (i) NKT cells in the experiments shown in Fig 2G–2I, Fig 4D and 4E, and Fig 4J and 4K. Cells were identified by the following cell surface markers: iNKT cells (CD1d-tet+CD3+), NK cells (NK1.1+CD3-CD1d-tet-TCRγδ-), γδ T cells (TCRγδ+CD3+CD1d-tet-), CD4 T cells (CD4+CD3+CD1d-tet-TCRγδ-CD8-), and CD8 T cells (CD8+CD3+CD1d-tet-TCRγδ-CD4-). (C) Gating strategy for defining lymphocytes using NK1.1 and CD3 to identify NKT cells in the experiments shown in Fig 2D–2F and Fig 4A. For these experiments, cells were identified by the following cell surface markers: γδ T cells (TCRγδ+CD3+), NK cells (NK1.1+TCRγδ-CD3-), NKT cells (NK1.1+CD3+TCRγδ-), CD4 T cells (CD4+CD3+TCRγδ-NK1.1-CD8-), and CD8 T cells (CD8+CD3+CD1d-tet-TCRγδ-NK1.1-CD4-). (TIF) [file pone.0167693.s006.tif]

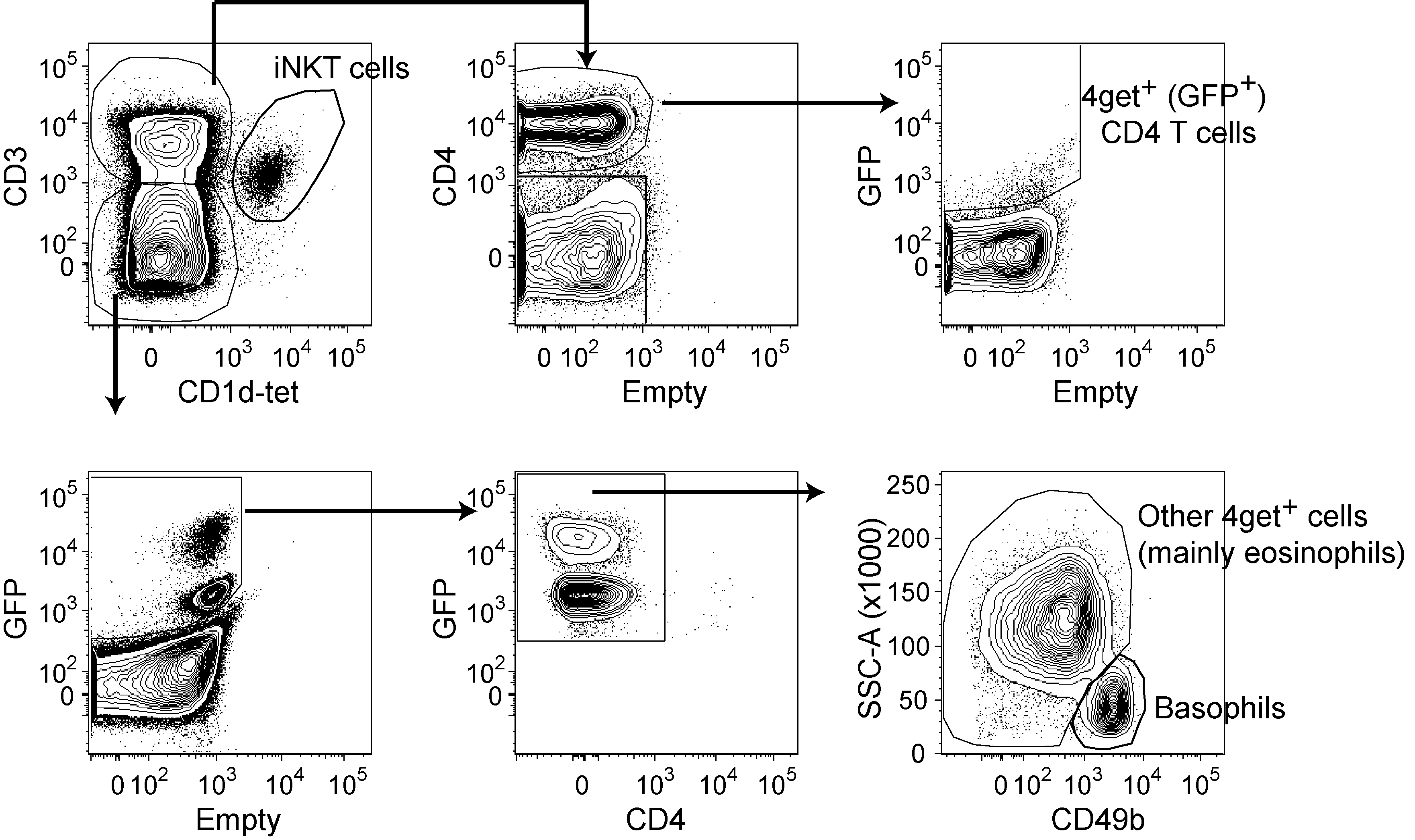

Supplement: S7 Fig — Gating strategy for 4get reporter+ CD4 T cells and basophils in the lungs of 4get/KN2 reporter mice as shown in Fig 2A–2C. Cells were identified by using the following markers: 4get+(GFP+) CD4 T cells (GFP+CD4+CD3+CD1d-tet-) and basophils (GFP+CD49b+SSCloCD3-CD1d-tet-CD4-). Basophils and eosinophils are constitutively 4get+ [31]. The gating strategy shown is from Myd88fl/+ Il44get/KN2 Mcpt8Basopho8/+ mice. Mcpt8Basopho8 mice express both YFP and Cre in basophils [61]. Both GFP from 4get reporter and YFP from Basopho8 reporter were read using the same filter/channel on the flow cytometer, and additional markers were used to distinguish basophils as described above. (TIF) [file pone.0167693.s007.tif]

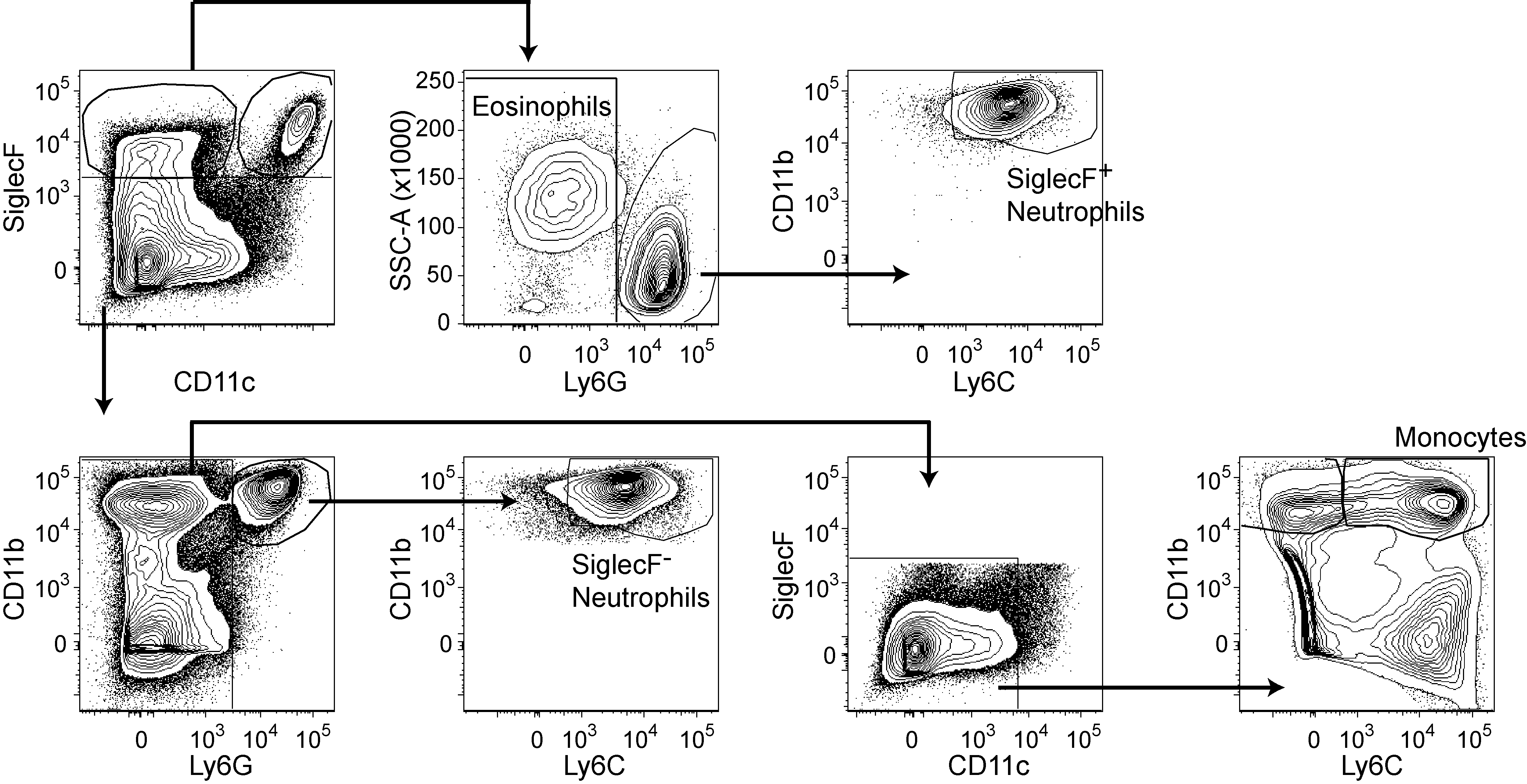

Supplement: S8 Fig — Gating strategy for identifying non-lymphocyte populations in the lung and BAL fluid in experiments shown in Fig 1B and Fig 3A–3C. Cells were identified by using the following cell surface markers: eosinophils (SiglecF+CD11b+CD11c-Ly6G-), neutrophils (Ly6G+Ly6C+CD11b+), and monocytes (Ly6C+CD11b+CD11c-/intSiglecF-Ly6G-). (TIF) [file pone.0167693.s008.tif]

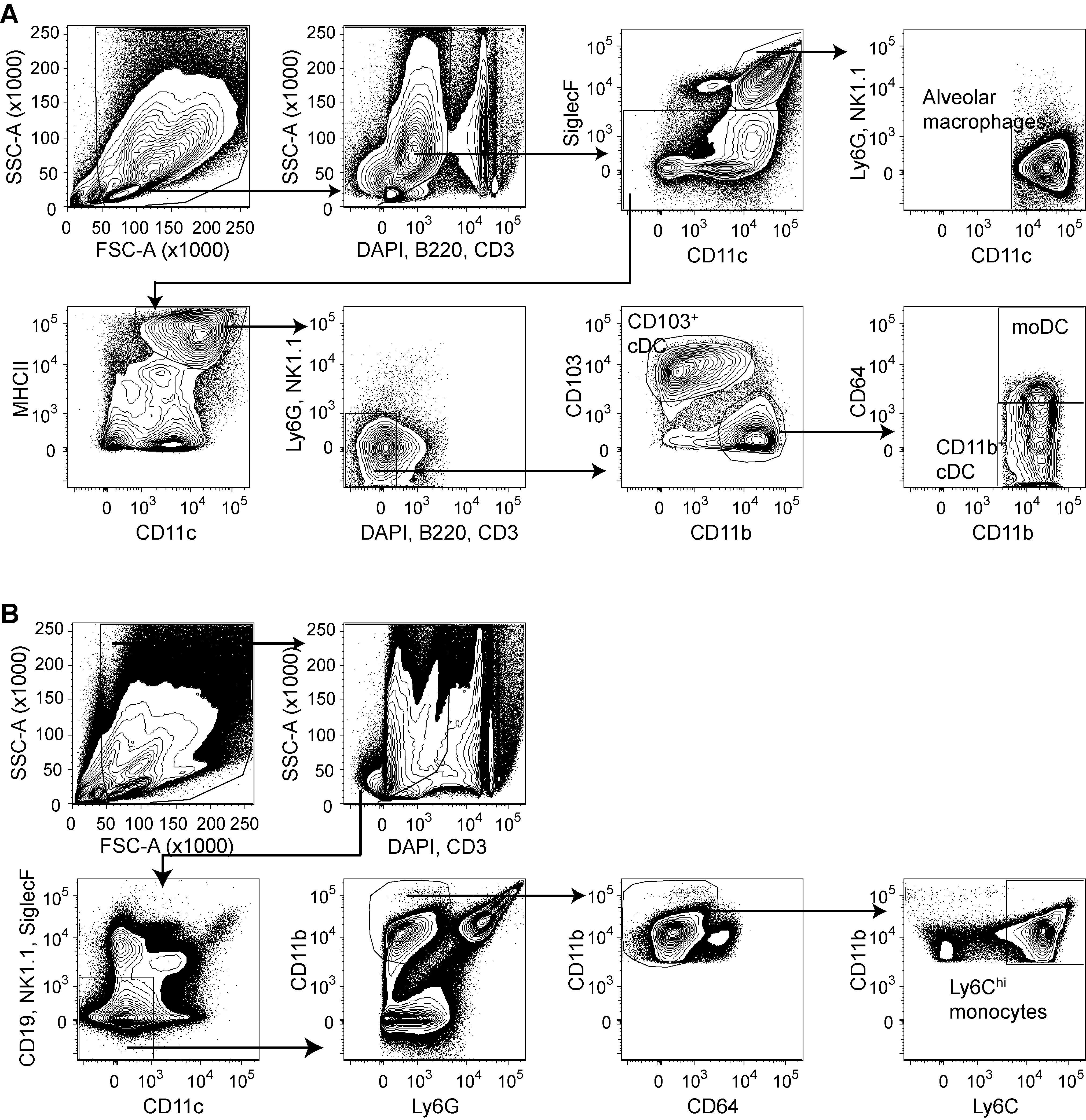

Supplement: S9 Fig — Gating strategy for experiments shown in For Figs 4H and 4I and 6F. (A) AMs, CD103+ cDCs, CD11b+ cDCs, and moDCs from CD11c-enriched cell suspensions were sorted as follows: AMs (SiglecF+CD11c+B220-CD3-NK1.1-Ly6G-), CD103+ cDCs (CD11c+MHCII+CD103+B220-CD3-NK1.1-Ly6G-SiglecF-) CD11b+ cDCs (CD11c+MHCII+CD11b+CD64-B220-CD3-NK1.1-Ly6G-SiglecF-), andmoDCs (CD11c+MHCII+CD11b+CD64+B220-CD3-NK1.1-Ly6G-SiglecF-). (B) Ly6Chi monocytes from CD11c-depleted lung cell suspension were sorted as follows: Ly6Chi CD11b+SiglecF-CD11c-CD19-CD3- CD64- Ly6G-NK1.1- (TIF) [file pone.0167693.s009.tif]

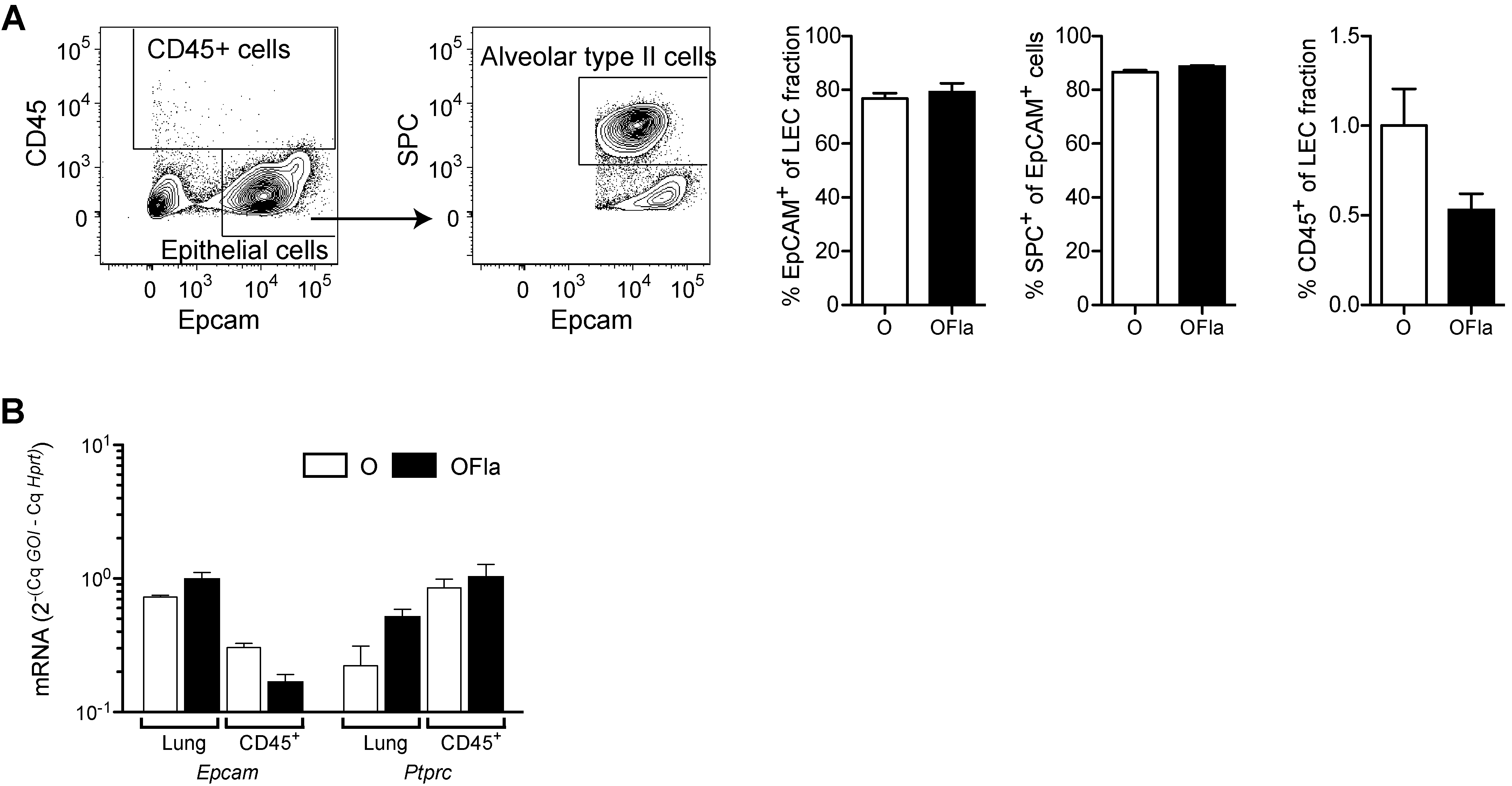

Supplement: S10 Fig — Cell enrichments were assessed after the LEC and CD45 cell separation from the lung. (A) Representative flow cytometry plots of LEC fraction stained with CD45, EpCAM, and SPC, and percentages EpCAM+ of LEC fraction, percentages SPC+ of EpCAM+ LEC fraction, and percentages of CD45+ of LEC fraction. (B) Epcam and Ptprc RNA analysis of CD45 fraction as compared to whole lung. Data contain 3–4 mice per group and are representative of two independent experiments. Error bars indicate mean +SD. (TIF) [file pone.0167693.s010.tif]
